# Oral Microbiome and CPT1A Function in Fatty Acid Metabolism in Oral Cancer

**DOI:** 10.3390/ijms252010890

**Published:** 2024-10-10

**Authors:** Zeba Praveen, Sung-Weon Choi, Jong Ho Lee, Joo Yong Park, Hyun Jun Oh, Ik Jae Kwon, Jin Hee Park, Mi Kyung Kim

**Affiliations:** 1 Department of Cancer Biomedical Science, Graduate School of Cancer Science and Policy, National Cancer Center, Goyang 10408, Republic of Korea; 2017102@ncc.re.kr; 2 Oral Oncology Clinic, Research Institute and Hospital, National Cancer Center, 323 Ilsandong-gu, Goyang-si 10408, Gyeonggi-do, Republic of Korea; choiomfs@ncc.re.kr (S.-W.C.); leejongho@ncc.re.kr (J.H.L.); slowp@ncc.re.kr (J.Y.P.); victor.oh@ncc.re.kr (H.J.O.); 3 Department of Oral and Maxillofacial Surgery, College of Dentistry, Seoul National University, 101 Daehak-ro, Jongno-gu, Seoul 03080, Republic of Korea; ijkwon@snu.ac.kr; 4 Cancer Epidemiology Branch, Division of Cancer Epidemiology and Prevention, National Cancer Center, 323 Ilsandong-gu, Goyang-si 10408, Gyeonggi-do, Republic of Korea

**Keywords:** oral microbiota, oral cancer, machine learning, SCFAs, CPT1A, disease-free survival, overall survival

## Abstract

The oral microbiome is crucial for human health. Although oral dysbiosis may contribute to oral cancer (OC), the detailed relationships between the microbiome and OC remain unclear. In this case-control study, we aimed to elucidate the connection between the oral microbiome and mechanisms potentially involved in oral cancer. The study analyzed 1022 oral saliva samples, including 157 from oral cancer patients and 865 from healthy controls, using 16S ribosomal RNA (16S rRNA) sequencing and a Light Gradient Boosting Machine (LightGBM) model to identify four bacterial genera significantly associated with oral cancer. In patients with oral cancer, the relative abundance of *Streptococcus* and *Parvimonas* was higher; *Corynebacterium* and *Prevotella* showed decreased relative abundance; and levels of fatty acid oxidation enzymes, including Carnitine palmitoyltransferase 1A (CPT1A), long-chain acyl-CoA synthetase, acyl-CoA dehydrogenase, diacylglycerol choline phosphotransferase, and H+-transporting ATPase, were significantly higher compared to controls. Conversely, healthy controls exhibited increased levels of short-chain fatty acids (SCFAs) and CD4+T-helper cell counts. Survival analysis revealed that higher abundance of *Streptococcus* and *Parvimonas*, which correlated positively with interleukin-6, tumor necrosis factor-alpha, and CPT1A, were linked to poorer disease-free survival (DFS) and overall survival (OS) rates, while *Prevotella* and *Corynebacterium* were associated with better outcomes. These findings suggest that changes in these bacterial genera are associated with alterations in specific cytokines, CPT1A levels, SCFAs in oral cancer, with lower SCFA levels in patients reinforcing this link. Overall, these microbiome changes, along with cytokine and enzyme alterations, may serve as predictive markers, enhancing diagnostic accuracy for oral cancer.

## 1. Introduction

Oral cancer is common globally, with an estimated 377,713 cases reported in 2020 [1]. Established risk factors for oral cancer include smoking, alcohol use, human papillomavirus infection, and sunlight exposure [2,3]. Associations between the oral microbiome and oral cancer have increasingly attracted attention; however, numerous knowledge gaps persist. Understanding the intricate process of oral cancer progression, identifying accurate oncological biomarkers, and implementing targeted therapies at an early stage are essential for effective oral cancer management [4,5].

The oral cavity comprises different hard tissues and mucosal structures and has a diversified microbial abundance; it is home to over 700 bacterial species, making it home to a significant microbial community and second only to the gastrointestinal tract [6]. This vast microbial community is vital for oral and systemic health, and dysbiosis is associated with inflammation and diseases, including oral cancer [4,7]. Bacteria such as *Streptococcus* and *Fusobacterium nucleatum* induce inflammation, suppress immunity, and alter cell signaling, thereby promoting oral cancer [8,9,10]. Conversely, *Lactobacillus plantarum* elicits anticancer effects via immune modulation and metabolite-driven tumor inhibition in oral cancer [11]. Therefore, oral-microbiome modulation represents a promising route for cancer prevention, therapeutic interventions, and overall oral health.

Metabolites produced by the oral microbiome, such as short chain fatty acids (SCFAs) and long-chain fatty acids, significantly influence cancer [12]. Some oral bacteria, such as Streptococcus and Fusobacterium, may reduce levels of short-chain fatty acids (SCFAs) and elevate tumor necrosis factor-alpha (TNF-α) and interleukin-6 (IL-6), potentially promoting cancer cell proliferation [13]. CD4+T-helper cells play a critical role in orchestrating immune responses, and their downregulation has been observed in various cancers, including colorectal [14] and pancreatic cancers [15]. In these contexts, the gut microbiota modulates T cell trafficking and contributes to immune evasion and tumor progression. Similarly, the downregulation of CD4+ T cells in oral cancer suggests a significant mechanism of immune suppression, highlighting the potential for targeting both T cell pathways and microbiome interactions in therapeutic interventions. Moreover, emerging evidence shows that specific gut bacteria can profoundly affect the production of carnitine palmitoyltransferase 1 (CPT1), an essential enzyme in mitochondrial fatty acid oxidation (FAO) that facilitates the generation of adenosine triphosphate (ATP), the primary energy source for cells [16]. Disruption of FAO may promote the progression of oral cancer [17,18]. Certain oral bacteria, such as Porphyromonas gingivalis, are potential cancer-causing agents that induce oxidative stress via oxidative stress-responsive kinase 1 (OXSR1) and DNA damage, whereas other bacteria, such as Lactobacillus, act as antioxidants that potentially suppress colon cancer [19,20].

Understanding the association between bacteria and oral cancer is hindered by variations in research methods and inconsistent findings across studies involving different cancer subtypes and stages. We investigated whether a synergistic interaction between the oral microbiome and underlying metabolic pathways, particularly involving enzymes such as carnitine O-palmitoyltransferase 1 (CPT1A), plays a key role in oral cancer. Our study also explored the plausible association of the oral microbiome with enzymes and cytokines related to fatty acid metabolism, oxidative stress, and immune responses, all of which are considered crucial for the initiation of oral cancer. Notably, survival analysis indicated that elevated levels of Streptococcus and Parvimonas, which correlated positively with interleukin-6, tumor necrosis factor-alpha, and CPT1A, were linked to poorer disease-free and overall survival outcomes. Additionally, we found that specific metabolites, such as short-chain fatty acids (SCFAs), were downregulated in oral cancer, suggesting a significant impact of the oral microbiome on these metabolic processes and their potential influence on patient survival.

## 2. Results

### 2.1. Demographic Characteristics of Oral Cancer Patients and Controls

A total of 1022 participants were included in the study, comprising 157 oral cancer patients and 865 healthy controls. The study involved two datasets: the discovery dataset with 637 participants (104 oral cancer cases and 533 controls) and the validation dataset with 385 participants (53 oral cancer cases and 332 controls). Detailed demographic characteristics of both the oral cancer-patient and control groups are presented in Table 1 and Figure S1.

### 2.2. Contrasting Oral Microbiome Compositions in Oral Cancer Patient and Control Groups: Taxonomic Analyses and Identification of Cancer-Associated Genera

We identified 48 phyla and 1704 genera across all study subjects. The dominant phyla were (relative abundance > 1%) *Proteobacteria*, *Bacteroidetes*, and *Firmicutes*, which accounted for over 90% of the bacterial population (Supplementary Figure S2A). The average number of reads per sample was 40,000, with a minimum threshold of 20,000 reads per sample. Patients with oral cancer exhibited significantly higher alpha diversity, as demonstrated by Observed OTU, Chao1, and Shannon indices, compared to the controls. In contrast, beta diversity, assessed through PCoA, was based on weighted and unweighted UniFrac distances, and patients with oral cancer showed a significantly higher alpha bacterial diversity compared to the controls. However, beta diversity analysis did not reveal any significant differences between the groups. This analysis does not assess the risk for cancer; rather, it provides information on associations with cancer versus control (Figure S2B–D). To examine fluctuations in oral microbiota between cancer patients and controls, we performed a linear discriminant analysis at the genus level. The analysis revealed that *Streptococcus* and *Parvimonas* were associated with an increased risk of oral cancer, while *Corynebacterium* and *Prevotella* were significantly associated with a reduced risk (Figure S2E). Furthermore, using Venn diagram analysis, we assessed oral microbiota in oral cancer patients and control participants, which revealed 740 distinct overlapping bacterial genera (Figure S2F).

### 2.3. Machine-Learning Analysis Identified Microbial Biomarkers for Oral Cancer Risk: LightGBM Model Accuracy and Microbiome Associations

**Figure 1:**
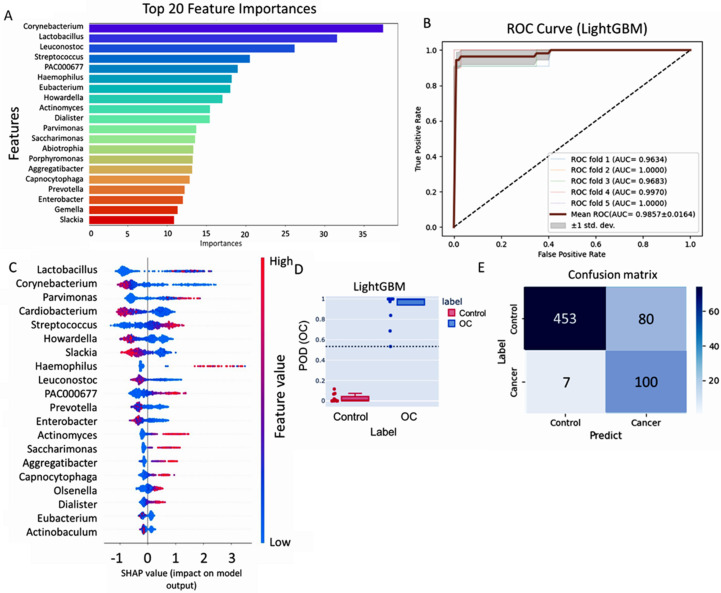
Oral microbiota-based prediction of oral cancer (OC) using the Light Gradient Boosting Machine learning (LightGBM) model. Machine-learning model training strategy: 5-fold cross-validation of the LightGBM model in predicting oral cancer (OC) dataset. (**A**) Feature importance analysis of the top 20 features contributing to the LightGBM model performance in discovery dataset (n = 637) Color Gradient: The colors range from deep blue at the top (for the highest importance) through green and yellow to red at the bottom (for lower importance). (**B**) Receiver Operating Characteristic (ROC) curve analysis showing the relationship between the true positive rate (TPR) and the false positive rate (FPR) of the LightGBM model. The area under the Receiver Operating Characteristic curve (ROC curve) represents the performance of the model in distinguishing between positive and negative samples in top 20 feature importance in the validation dataset (n = 385). (**C**) The utilization of SHapley Additive exPlanations (SHAP) values has unveiled a comprehensive elucidation of the output generated by the machine learning model. This method offers a localized interpretation for individual predictions, accomplishing this by assigning proportional contributions of features to the ultimate prediction outcome. (**D**) The Probability of Detection Index (POD) in Light Gradient Boosting Machine Learning indicates the likelihood of classifying samples into cancer and control, with values ranging from 0 to 1. Higher values closer to 1 suggest a greater chance of classifying a sample as cancer. (**E**) The fold confusion matrix evaluated the LightGBM model performance with actual and predicted labels in the discovery dataset.

**Figure 2:**
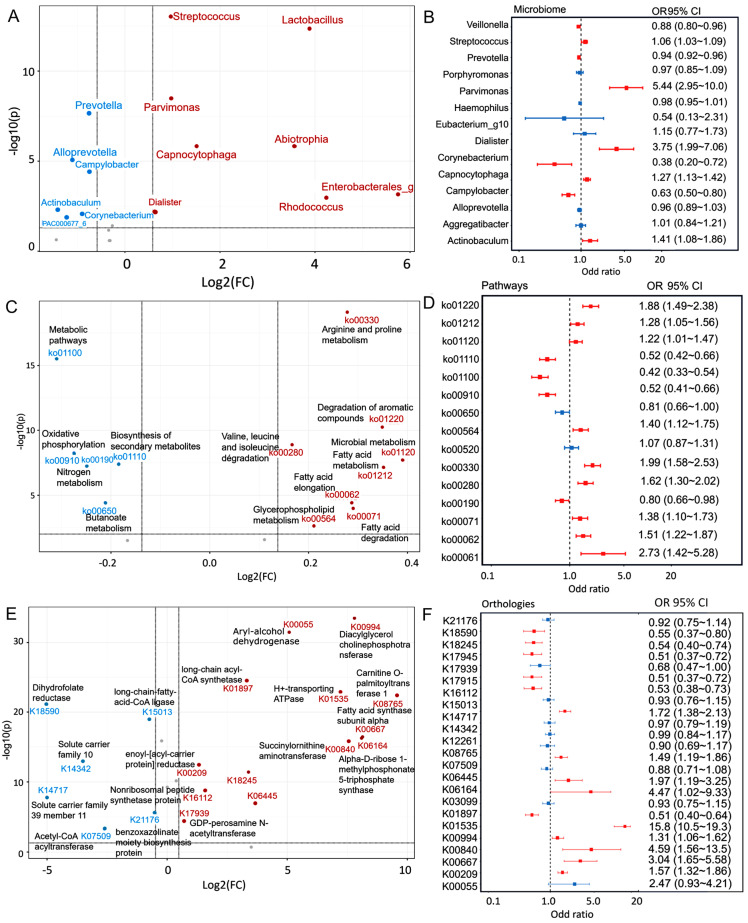
(**A**,**C**,**E**) Volcano plot using the results of a fold change of 20 microbiomes, orthologies, and pathways in OC, compared to controls. Red dots indicate significant microbiome, orthologies, and pathways with |log2FC| > 0.5; blue dots indicate non-significant microbiome, orthologies, and pathways with |log2FC| > 0.5; and gray dots indicate non-significant microbiome, orthologies, and pathways with |log2FC| < 0.5. (**B**,**D**,**F**) A graphical representation of a forest plot displaying odds ratios and their corresponding 95% confidence intervals (95% CI) reveals the results of a multivariate logistic regression analysis involving the continuous scale of microbiome, orthologies, and pathways. In the odds ratio plot, the red line indicates significant microbiome, orthologies, and pathways; blue lines indicate non-significant microbiome, orthologies, and pathways. OC, oral cancer; CI, confidence interval.

Machine-learning techniques can identify microbial taxa that may serve as oral cancer biomarkers. Utilizing the LightGBM model, we achieved a high accuracy level in predicting oral cancer, with an F1 score of 0.90, a sensitivity of 0.96, a precision of 0.98, an AUC of 0.98, and an overall accuracy of 0.97 (Table S1). The performance was evaluated using a five-fold cross-validation approach. Top-feature importance analysis of the model revealed significant contributors to cancer prediction performance via ROC analysis (Figure 1A–E, Figure S3). The analysis of 20 selected microbial taxa revealed that higher abundances of *Streptococcus* (fold-change = 1.964, *p* = 2.01 × 10^−16^) and *Parvimonas* (fold-change = 1.951, *p* = 2.01 × 10^−14^) were linked to an increased cancer risk, whereas *Corynebacterium* (fold-change = 0.543, *p* = 2.91 × 10^−3^) and *Prevotella* (fold-change = 0.590, *p* = 3.86 × 10^−11^) were linked to a reduced cancer risk (Figure 2A, Table S2). An odds ratio plot was constructed to assess the relationships for each genus using logistic-regression analysis. Our results revealed higher relative abundances of *Streptococcus* and *Parvimonas* and a lower relative abundance of *Corynebacterium* and *Prevotella* in the oral cancer-patient group than in the control group (Figure 2B, Table S3).

### 2.4. Integration of the Microbiome and Functional-Pathway Analysis Revealed Potential Biomarkers and Associations in Oral Cancer

In this study, we identified 14,860 KEGG orthologs (KOs) and 446 pathways using PICRUSt and data from 1022 participants. This analysis revealed 24 significantly different KOs between the groups. CPT1A, acyl-CoA dehydrogenase, long-chain acyl-CoA synthetase, diacylglycerol choline phosphotransferase, and H+-transporting ATPase were associated with an elevated cancer risk (Figure 2C,D, Tables S4 and S5). A parallel analytical approach was used to predict outcomes associated with 15 specific pathways. Fatty acid metabolism (ko01212) and biosynthesis (ko00061) were significantly associated with oral cancer progression (Figure 2E,F, Tables S6 and S7).

**Figure 3:**
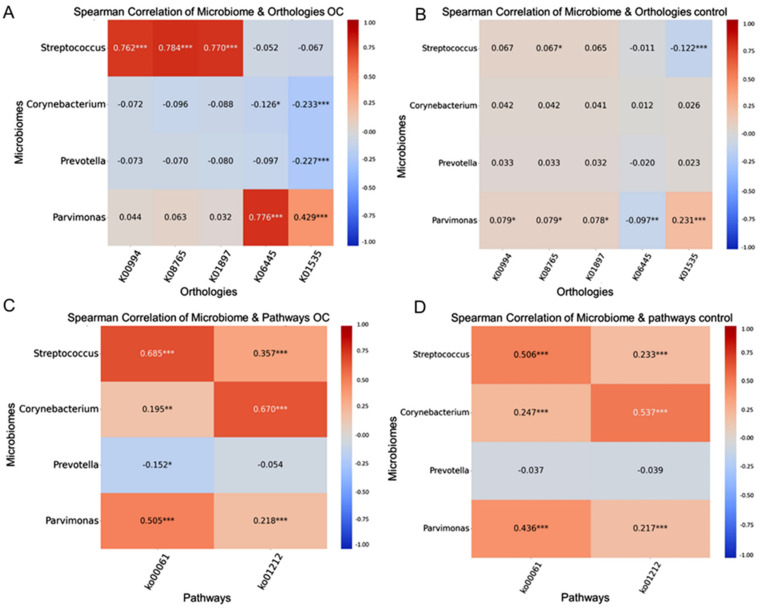
Spearman correlation heatmap between microbiome and function data: (**A**,**B**) correlation between four genera and five orthologs (Carnitine O-palmitoyltransferase 1(K08765), Acyl-CoA dehydrogenase (K06445), Long-chain acyl-CoA synthetase (K01897), Diacylglycerol choline phosphotransferase (K00994), and H+transporting ATPase (K01535)) in oral cancer and control group; (**C**,**D**) correlation between four genera and two pathways (Fatty acid biosynthesis (ko00061) and Fatty acid metabolism (ko01212)) in cancer and control. Red indicates a positive correlation, while blue indicates a negative correlation. * Significant correlation at the *p* < 0.05 level. ** more significant correlation at the *p* < 0.01 level. *** highly significant correlation at the *p* < 0.001 level. (The color chart range has been set from −1.00 to 1.) OC, oral cancer.

We explored the relationship between 20 specific microbiomes and 24 KOs using Spearman’s rank correlation coefficients. In patients with oral cancer, enzymes such as CPT1A (K08765), acyl-CoA synthetase (K01897), and diacylglycerol choline phosphotransferase (K00994) correlated positively with *Streptococcus*, whereas acyl-CoA dehydrogenase (K06445) and H+-transporting ATPase (K01535) correlated positively with *Parvimonas* (Figure 3A,B; Figure S4A,B). Fatty acid degradation (ko00061) was significantly and positively correlated with *Streptococcus* and *Parvimonas* in the oral cancer group. In contrast, fatty acid metabolism (ko01212) showed greater correlations with *Streptococcus*, *Parvimonas*, and *Corynebacterium* in the oral cancer group than in the control group (Figure 3C,D; Figure S4C,D).

### 2.5. Microbiome Associations with Disease-Free and Overall Survival in Oral Cancer

We assessed the association between specific oral microbiota and patient survival outcomes. Our findings revealed significant links between certain taxa and both disease-free survival (DFS) and overall survival (OS) rates. Notably, Streptococcus and Parvimonas were associated with worse outcomes; Streptococcus exhibited an independent increased risk for disease recurrence and mortality (HR: 2.85 for DFS, 5.76 for OS), while Parvimonas also indicated an elevated risk (HR: 2.17 for DFS, 2.90 for OS). In this multivariate analysis, we accounted for potential confounding factors, particularly disease stage, which is crucial for accurate cancer prognosis, as advanced stages may correlate with specific microbiota profiles and poorer survival outcomes.

Conversely, *Prevotella* and *Corynebacterium* were linked to reduced recurrence and mortality risk, demonstrating protective effects (HR: 0.28 for DFS, 0.30 for OS for *Prevotella*; HR: 0.26 for DFS, 0.36 for OS for *Corynebacterium*). These results underscore the prognostic value of the oral microbiome in oral cancer, highlighting how specific bacterial taxa can influence disease progression and patient survival, and are presented in Table 2.

**Figure 4:**
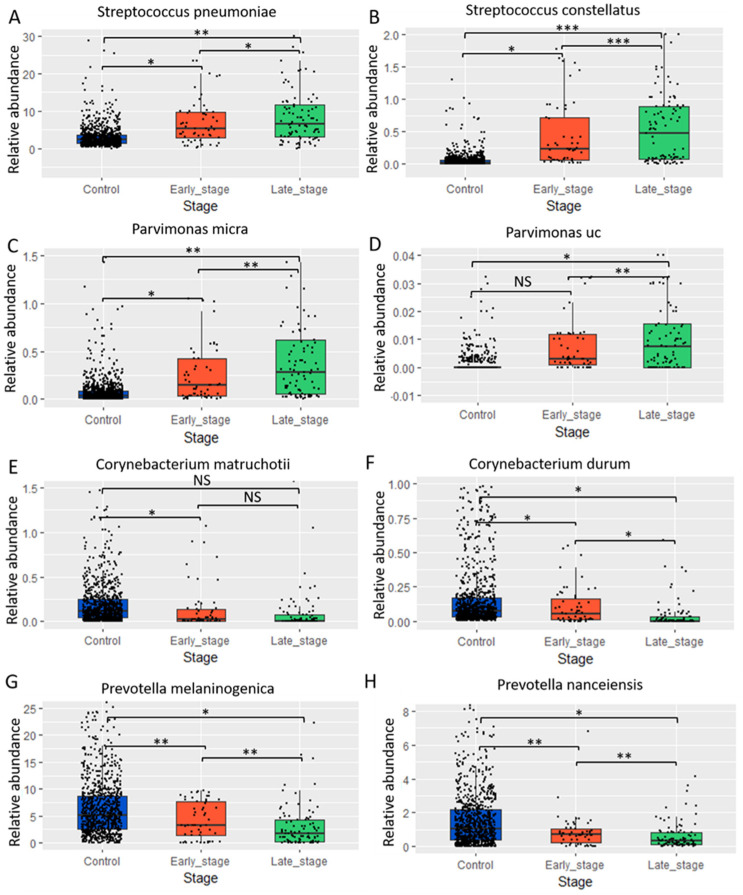
Relative abundance of various bacterial species in control, early stage, and late stage in oral cancer saliva microbiome: (**A**) *Streptococcus pneumoniae*, (**B**) *Streptococcus constellatus*, (**C**) *Parvimonas micra*, (**D**) *Parvimonas uncultured (uc)*, (**E**) *Corynebacterium matruchotii*, (**F**) *Corynebacterium durum*, (**G**) *Prevotella melaninogenica*, (**H**) *Prevotella nanceiensis*. Statistical significance * *p* < 0.05, ** *p* < 0.01, *** *p* < 0.001, NS: not significant.

After analyzing bacterial genera, we further assessed the relationship between clinicopathological stage and bacterial species, to gain a deeper understanding of the oral microbiome. We examined the relative abundance of bacterial species in control, early-stage, and late-stage samples. In the genera *Streptococcus* and *Parvimonas*, the species *Streptococcus pneumoniae*, *Streptococcus constellatus*, *Parvimonas (uncultured)*, and *Parvimonas micra* exhibited a significant increase in relative abundance at the late stage compared to both the early-stage and control samples, with significant differences noted across all three stages (Figure 4A–D). Conversely, the genera *Corynebacterium* and *Prevotella*, specifically *Corynebacterium matruchotii*, *Corynebacterium durum*, *Prevotella melaninogenica*, and *Prevotella nanceiensis*, showed a marked increase in abundance in control and early-stage samples relative to the late stage of the oral cancer microbiome (Figure 4E–H).

### 2.6. Oral Microbiome Modulation of Cytokine and Immune Pathways: Implications for Cancer Onset

Combining microbiome analysis, IHC, and ELISA aims to reveal microbial and cellular roles in oral cancer. Microbiome analysis examines bacterial impacts on immunity and metabolites, IHC localizes key proteins, and ELISA quantifies cytokines. Together, these methods elucidate molecular mechanisms linking the oral microbiome to cancer progression.

**Figure 5:**
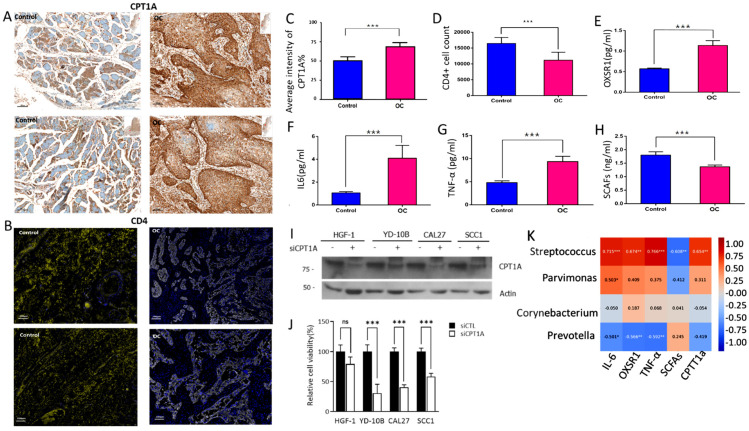
Impact of CPT1A siRNA treatment on cell viability, CPT1A depletion, immunohistochemical and biomarker analysis in oral cancer. (**A**) Representative images of immunohistochemistry of CPT1A protein detected from oral tissues. Stained tissues are shown at 100× magnification. Scale bar represents 100 μm. (**B**) IHC staining of control and OC tissues; the staining is visualized using a yellow color (Opal 480 yellow with scale bar: 200 μm) and DAPI in OC, oral cancer. (**C**) Quantified intensity of CPT1A in OC. Error bars indicate the mean ± SEM for three independent experiments. (**D**) Quantified CD4+T-helper cell counts. (**E**) Oxidative Stress Responsive 1 (OXRS1). (**F**) Human plasma interleukin-6 levels (IL6). (**G**) Tumor necrosis factor-alpha (TNFα). (**H**) Short-chain fatty acid (SCFA) concentrations in oral saliva. OC: oral cancer. (**I**) Western blot analysis showing the depletion of CPT1A by siRNA treatment in normal and oral cancer cells. The data are representative of at least three independent experiments. (**J**) HGF-1, YD-10B, CAL27 and SCC1 cells were reverse-transfected with either siCTL or siCPT1A; after 48 h, cell viability of HGF-1, YD-10B, CAL27 and SCC1 cells was analyzed using MTT assay. (**K**) Spearman correlation heatmap for four specific microbiomes with cytokines and enzyme in oral cancer. (The color chart range has been set from −1.00 to 1.00.) Statistical significance * *p* < 0.05, ** *p* < 0.01, *** *p* < 0.001, ns: not significant.

IHC and ELISA experiments were conducted to further analyze the significant pathways identified (K08765, K06445, K01897, K00994, and K01535). Oral cancer tissues showed significantly elevated levels of the CPT1A protein; an increased presence of T-helper cell counts (CD4+); and higher levels of OXSR1, IL-6, and TNF-α. SCFAs production by oral microbial communities were lower in the oral cancer group than in the control group (Figure 5A–H).

MTT assays demonstrate that siCPT1A treatment effectively reduces CPT1A protein expression across four cell lines (HGF-1, YD-10B, CAL27, and SCC1), while Actin levels remain consistent, confirming equal protein loading (Figure 5I). The bar graph indicates that siCPT1A treatment significantly decreases cell viability in YD-10B, CAL27, and SCC1 cells (highly significant in YD-10B and SCC1, extremely significant in CAL27), but not in HGF-1 cells. These findings suggest that CPT1A is crucial for the viability of YD-10B, CAL27, and SCC1 cell lines, but not for HGF-1, indicating varying dependence on CPT1A among these cell lines (Figure 5J). Additionally, MTT assays revealed that CPT1A knockdown affects the survival of both cancerous and normal cells. Further exploration of the relationship between specific microbiomes and cytokines/enzymes revealed that *Streptococcus* correlated positively with IL-6, TNF-α, CPT1A, and OXSR1 levels; *Parvimonas* correlated positively with IL-6 alone; and *Prevotella* correlated negatively with IL-6, OXSR1, and TNF-α. These correlations suggest that the oral microbiome may significantly influence cytokine and CPT1A levels, thereby affecting cellular functions and survival (Figure 5K).

## 3. Discussion

This case-control study identified significant associations between specific members of the oral microbiome and the metabolic pathways involved in oral cancer. Patients with oral cancer exhibited notable changes in enzymes critical for fatty acid metabolism, particularly CPT1A, while healthy controls had higher levels of short-chain fatty acids (SCFAs) and CD4+T-helper cell counts. In contrast, oral cancer patients demonstrated elevated concentrations of key markers, including CPT1A, IL-6, OXSR1, and TNF-α, reflecting substantial alterations in metabolic and inflammatory pathways. Survival analysis revealed that higher levels of *Streptococcus* and *Parvimonas*, positively correlated with interleukin-6, tumor necrosis factor-alpha, and CPT1A, were linked to poorer disease-free and overall survival, whereas increased levels of *Prevotella* and *Corynebacterium* were associated with better survival outcomes.

Reports on the association between the oral microbiome and oral cancer are limited [21]; the role of CPT1A as a regulator of fatty acid metabolism with relevance to the oral microbiome and oral cancer risk has rarely been investigated. In this study, we elucidated associations between the oral microbiome, cellular enzymes, and cytokines in oral cancer, in detail. We observed that the levels of *Corynebacterium* and *Prevotella* decreased in relative abundance significantly in patients with oral cancer, whereas the levels of *Streptococcus* and *Parvimonas* increased in relative abundance significantly. Our findings are consistent with those of earlier reports suggesting that bacterial imbalance may upset the complex equilibrium of oral microbiome–host relationships and cause oral cancer [22,23,24,25,26].

The findings of this study indicate that changes in fatty acid metabolism enzymes may contribute to the development of oral cancer. Specific bacterial species, such as Corynebacterium glutamicum and Prevotella intermedia, have been linked to fatty acid metabolism through β-oxidation and energy generation, as documented in the KEGG database [27,28]. Additionally, an upregulation of short-chain fatty acids (SCFAs) was observed in healthy controls. Previous studies have highlighted the potential role of SCFAs from gut microbiota in cancer treatment by influencing apoptosis, cell cycle arrest, and metabolism. SCFAs may also enhance the efficacy of conventional therapies and reduce drug resistance. Future research should focus on optimizing SCFA-based treatments and investigating their role in personalized cancer care [29].

Moreover, other studies have shown a close association between Streptococcus and Parvimonas with the production of various SCFAs and bile acids [30,31], which differ from the fatty acids metabolized by CPT1A in the mitochondria of various cancers [17,32]. CD4+ T-helper cell counts were lower in oral cancer patients compared to healthy controls, suggesting potential immune dysregulation. This finding aligns with research indicating that microbiota-derived SCFAs support CD4+ T cell differentiation and function. Therefore, disruptions in microbiome composition or SCFA signaling in oral cancer patients may contribute to impaired immune responses, potentially influencing disease progression and immune surveillance [33,34]. Furthermore, the results demonstrated a correlation between alterations in the oral bacterial community, increased lipid metabolism, and oxidative stress in oral cancer. These changes are linked to variations in CPT1A enzyme levels and the production of key metabolites, such as fatty acids. Together, these insights point to potential lipid-related mechanisms in oral cancer development, underscoring the importance of exploring oral microbiome imbalances for a deeper understanding and improved diagnosis of the disease.

**Figure 6:**
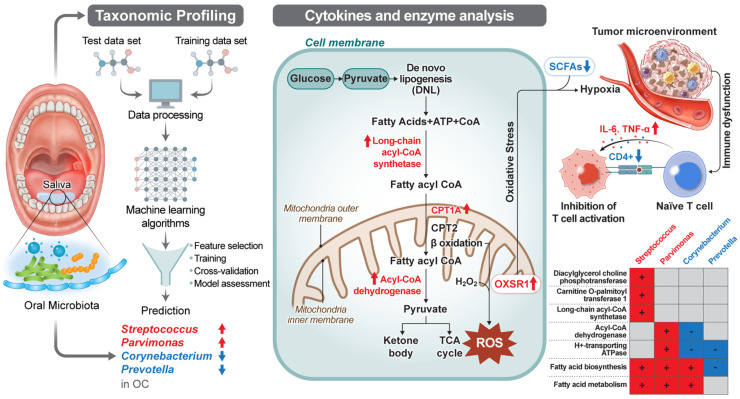
Exploring the impact of oral microbiota on carnitine O-palmitoyltransferase 1A (CPT1A) function in fatty acid metabolism and its potential immunomodulatory effects in oral cancer. The oral microbiome may modulate the upregulation of CPT1A, Oxidative Stress Responsive Kinase 1 (OXSR1), Interleukin-6 (IL-6), and Tumor Necrosis Factor-alpha (TNF-α) in oral cancer patients, while concurrently influencing the downregulation of short-chain fatty acids. Red: increased, blue: decreased.

Our findings highlight the crucial role of the oral microbiome in regulating key metabolic enzymes, such as CPT1A and OXSR1, as well as cytokines like TNF-α and IL-6 in oral cancer. Additionally, we observed the downregulation of specific metabolites, including SCFAs and CD4+T-helper cell counts, in oral cancer (Figure 6). These molecules can trigger distinct immune responses and pathways linked to carcinogenesis [35]. Our results are consistent with those of previous studies, in which oral bacteria were found to be associated with tumor metastasis through vascular inflammation and barrier disruption [36,37]. Previously, FAO upregulation increased oxidative stress and potentially increased OXSR1 levels [38]. Here, we demonstrated that CPT1A knockdown did significantly influence the survival of cancerous or normal cells. Previous data emphasized the significance of CPT1A in cancer cell proliferation, metastasis, and induced tumor senescence via FAO regulation [35,37,39,40,41,42,43,44]. Our findings confirm that CPT1A is a lipid-metabolism regulator associated with oral cancer.

The study has several significant limitations that must be acknowledged. The sample diversity and size, with participants drawn from specific institutions in Korea, may limit the generalizability of the findings to broader populations. As a case-control study, it identifies associations between oral microbiome composition and oral cancer based on comparisons between cancer patients and healthy controls, but it does not establish causality or the directionality of these relationships. Variability in sample collection and handling, including timing and storage conditions, could introduce biases affecting the results. Technical constraints inherent in 16S rRNA sequencing, such as short read lengths and low throughput, may impact the accuracy and scalability of microbial identification. Although the LightGBM model demonstrated high accuracy, its performance is sensitive to parameter choices and potential overfitting, highlighting the need for validation in independent cohorts. The functional predictions and pathway analyses based on inferred data may not fully capture actual microbial functions. Furthermore, biological variability among individuals and ethical considerations related to informed consent add complexity to interpreting and generalizing the findings. Addressing these limitations in future research is essential for a more comprehensive understanding of the oral microbiome’s role in oral cancer [45].

## 4. Materials and Methods

### 4.1. Participant Characteristics

In this case-control study, we enrolled adult patients (age > 19 years) newly diagnosed with oral squamous cell carcinoma, covering oral-cavity regions, from the National Cancer Center, Korea, and Seoul National University Dental Hospital. Healthy controls were recruited from the cancer-screening cohort of the National Cancer Center of the Republic of Korea. This study consisted of 1022 participants. The discovery and validation datasets included 637 participants (104 with oral cancer and 533 controls) and 385 participants (53 with oral cancer and 332 controls), respectively. By dividing the study into these two groups, we can ensure that their findings are not only discovered but also validated, and also increase the credibility of the results. Among the characteristic parameters, the main clinical variables considered were age, sex, smoking status, drinking status, cancer stage, tumor extent (T stage), and lymph node involvement (N stage). Body Mass Index (BMI) was treated as a continuous variable and categorized into five groups: underweight (<18.5), normal (18.5 to 23), overweight (23 to 25), obese (≥25), and unknown. Smoking status was classified into four groups: nonsmokers, current smokers, ex-smokers, and unknown. Similarly, drinking status was categorized as nondrinking, current drinking, ex-drinking, and unknown. Oral cancer was staged as T1, T2, T3, and T4, while lymph node involvement was classified as N0, N1, N2, and N3. The study was ethically approved by the National Cancer Center, Korea (IRB approval numbers NCC2019-0050, NCC2019-0116, and CRI15017), and written informed consent was obtained from all participants.

### 4.2. Saliva and Blood Sample Collection

Baseline saliva (unstimulated whole) samples from participants were meticulously collected following a 1 h fasting period, to minimize variability in saliva composition due to recent food and drink intake. These samples were then carefully stored in 1.5 mL tubes at −80 °C, ensuring their preservation for subsequent analysis. Blood samples were drawn from their antecubital veins into BD Vacutainer K2 EDTA tubes after a 12 h fast and centrifuged at 3000 rpm for 20 min at 4 °C. The resulting plasma, buffy coat, and red blood cell samples were stored at −80 °C. This was performed in compliance with the Declaration of Helsinki ethical principles for medical research involving human subjects

### 4.3. Oral Microbiome Characterization Based on 16S rRNA Gene Amplification and Sequencing

A substantial concentration of microbial DNA, precisely 70 ng/mL, was meticulously extracted from 500 µL of saliva samples employing the highly efficient Fast DNA Spin Kit (MP Biomedicals, Santa Ana, CA, USA) in accordance with the manufacturer’s instruction. From this, 20 ng/mL was used for further analysis. This advanced extraction method ensures the isolation of high-purity DNA, crucial for downstream molecular analyses and the exploration of the complex microbiome within the saliva. DNA quality and quantity were checked using a Qubit dsDNA BR Kit and a fluorometer (Life Technologies, Carlsbad, CA, USA). PCR amplification was performed using fusion primers targeting V4 regions of the 16S rRNA gene with the extracted DNA. For bacterial amplification, fusion primers of 515F (*5′-AATGATACGGCGACCACCGAGATCTACAC-XXXXXXXX-TCGTCGGCAGCGTCAGATGTGTATAAGAGACAG-GTGCCAGCMGCCGCGGTAA-3′*; underlining sequence indicates the target region primer) and 806R (*5′-CAAGCAGAAGACGGCATACGAGAT-XXXXXXXXGTCTCGTGGGCTCGG-AGATGTGTATAAGAGACAG-GGACTACHVGGGTWTCTAAT-3′*) were used. The Fusion primers are constructed in the following order: P5 (P7) graft binding, i5 (i7) index, Nextera consensus, sequencing adaptor, and target region sequence.

The amplifications were carried out under the following conditions: initial denaturation at 95 °C for 3 min, followed by 25 cycles of denaturation at 95 °C for 30 s, primer annealing at 55 °C for 30 s, and extension at 72 °C for 30 s, with a final elongation at 72 °C for 5 min. The PCR product was confirmed by using 1% agarose gel electrophoresis and visualized under a Gel Doc system (BioRad, Hercules, CA, USA). The amplified products were purified with the CleanPCR (CleanNA). Equal concentrations of purified products were pooled together and short fragments (non-target products) were removed with CleanPCR (CleanNA). The quality and product size were assessed on a Bioanalyzer 2100 (Agilent, Palo Alto, CA, USA) using a DNA 7500 chip. Mixed amplicons were pooled and the sequencing was carried out at CJ Bioscience, Inc. (Seoul, Korea), with Illumina iSeq Sequencing system (Illumina, San Diego, CA, USA) according to the manufacturer’s instructions. Bacteria were classified based on taxonomic data provided by EzBioCloud [46]. Poor-quality sequence reads of <80 base pairs (bp) or >2000 bp were excluded. Taxonomic analysis was performed using the USEARCH tool. The UPARSE algorithm was used to classify the reads into operational taxonomic units (OTUs) with 97% similarity. Single-end reads were clustered into OTUs using UCLUST and the cut-off numbers [47].

### 4.4. Functional Homology Inferences: Predicting Orthologs

The functional profile of the oral microbiome was constructed using the PICRUSt algorithm with EzBioCloud’s [46] microbiome taxonomic profiling (MTP). Sequencing reads were obtained using the EzBioCloud 16S MTP pipeline and matched to reference database entries. Functional profiles were annotated by multiplying the gene counts/OTU by the OTU abundance per sample, using the Kyoto Encyclopedia of Genes and Genomes (KEGG) database. The accuracy of each functional profile was analyzed using the nearest-sequenced taxon index.

### 4.5. Cell Lines

HGF-1, YD-10B, CAL27, and SCC1 are oral cancer cell lines, used in our studies due to their distinct characteristics and relevance to oral cancer research. HGF-1, CAL27, and SCC1 cells were cultured in Dulbecco’s Modified Eagle’s Medium (DMEM) with 10% fetal bovine serum (FBS), penicillin, and streptomycin, and maintained at 37 °C with 5% CO2. In contrast, YD-10B cells were grown in RPMI-1640 medium under the same conditions. HGF-1 provides a baseline for comparison with normal oral mucosal cells, while CAL27 and SCC1 are used to study cancer progression and therapeutic responses, given their origins from human oral squamous cell carcinomas. YD-10B, derived from another human oral cancer, helps assess experimental conditions and drug effects. These cell lines were selected to offer a comprehensive view of oral cancer biology and to facilitate the evaluation of new treatments.

### 4.6. Small-Interfering RNA (siRNA) Experiments

A negative-control siRNA and an siRNA targeting CPT1A mRNA were purchased from Genolution, Inc. (Seoul, Republic of Korea). The abovementioned siRNAs had the following sequences: siControl: *5′-CUCGUGCCGUUCCAUCAGGUAGUU-3′; siCPT1A: 5′-GACGUUAGAUGAAACUGAAUU-3′*.

### 4.7. 3-(4,5-Dimethylthiazol-2-yl)-2,5-diphenyltetrazolium bromide (MTT) Assay

The MTT (3-(4,5-dimethylthiazol-2-yl)-2,5-diphenyltetrazolium bromide) assay was performed to determine cell viability. HGF-1 (4 × 10^4^ cells/mL) and YD-10B, CAL27, SCC1 (3 × 10^4^ cells/mL) were grown in 96-well plates. After 24 h, the cells were reverse-transfected with either scrambled siRNA (siCTL) or siCPT1A-specific siRNA (siCPT1A) for 48 h. Following this incubation, MTT solution was added to each well at a final concentration of 5 mg/mL. After incubation at 37 °C for 6 h, the formazan pellets in each well were completely dissolved in 2-propanol (Merck, Rahway, NJ, USA), and the absorbance was measured using a VERSA Max Microplate Reader (Molecular Devices Corp., San Jose, CA, USA) at wavelengths of 540 nm and 650 nm.

### 4.8. Western Blotting

HGF-1, YD-10B, CAL27, and SCC1 cells were reverse-transfected with siCPT1A for 48 h. Subsequently, the cells were harvested in ice-cold RIPA lysis buffer (R0278; Sigma Aldrich, Seoul, Republic of Korea) containing protease and phosphatase inhibitors. Soluble lysate was isolated from each sample via centrifugation and quantified using the BCA Protein Assay Lit (Pierce, Thermo Fisher Scientific, Waltham, MA, USA). Proteins were resolved using sodium dodecyl-sulfate polyacrylamide gel electrophoresis and transferred to polyvinylidene fluoride or polyvinylidene difluoride membranes. The membranes were blocked with 5% skim milk and probed with a primary antibody against CPT1A (ab128568, Abcam, Cambridge, UK) and a secondary horseradish peroxidase-conjugated anti-mouse antibody (A90–116P; Bethyl Laboratories, Montgomery, TX, USA).

### 4.9. Immunohistochemistry (IHC) Analysis of CPT1A and CD4+T-Helper Cell Counts

A tissue microarray (TMA) was constructed using paraffin-embedded blocks from six tumor samples with a tissue array device (Beecher Instruments Inc., Sun Prairie, WI, USA). For each sample, 2 mm diameter core biopsies were extracted from the paraffin blocks. The TMA blocks were sectioned at a thickness of 3 μm and subsequently dried at 56 °C for 1 h. Immunohistochemical staining was performed using an automated Discovery XT instrument (Ventana Medical Systems, Tucson, AZ, USA) and the Chromomap DAB Detection Kit. Sections were deparaffinized, rehydrated, and washed with reaction Buffer (Ventana Medical Systems). Antigen retrieval was achieved through heat treatment in pH 6.0 citrate buffer (Ribo CC, Ventana) at 95 °C for 20 min. A primary antibody against CPT1A (15184-1-AP, Proteintech, dilution 1:200) was applied for 32 min at room temperature, followed by detection with an UltraMap anti-rabbit HRP secondary antibody for 16 min at room temperature. Images were captured using a Vectra Polaris system (PerkinElmer, Waltham, MA, USA). CPT1A protein expression was quantified using an H-score, calculated as follows: the percentage of tumor cells with weak staining intensity (+1) multiplied by 1, the percentage of cells with moderate staining intensity (+2) multiplied by 2, and the percentage of cells with strong staining intensity (+3) multiplied by 3. The H-score is the sum of these values: [1 × (% cells + 1) + 2 × (% cells + 2) + 3 × (% cells + 3)], resulting in a final CPT1A score ranging from 0 to 300 cells.

CD4+T-helper cell-count tissue samples were prepared as 4 μm thick sections using a microtome, deparaffinized, and rehydrated. Antigens were retrieved using Tris-EDTA and sodium citrate buffer (pH 6.0). After blocking peroxidases with 3% hydrogen peroxide, the samples were stained and scanned by PrismCDX Co., Ltd. (Gyeonggi-do, Korea), as per clinical protocols (Supplementary Protocol S1).

### 4.10. Antibodies and Reagents

Primary antibodies against CPT1A (ab128568) were purchased from Abcam (Cambridge, UK). Secondary antibodies against horseradish peroxidase-linked anti-mouse (A90–116P) were purchased from Bethyl Laboratories (Montgomery, TX, USA).

For IHC, primary antibodies against CPT1A (15184-1-AP) were purchased from Proteintech (Chicago, IL, USA).

### 4.11. Measuring Oral Microbial Signals, Including Cytokine Levels (OXSR1, CPT1A, SCFAs, IL6, and TNF-α)

Plasma oxidative stress was evaluated by performing enzyme-linked immunosorbent assays (ELISAs) using the OXSR1 ELISA Kit (abx382011; Abbexa Ltd., Cambridge, UK). Briefly, 100 µL saliva samples were aliquoted into 96-well plates and incubated at 37 °C. Detection Reagents A and B were then added to the plates, which were further incubated for 1 h at 37 °C. TMB substrate (90 µL) was added to each plate, followed by 50 µL of stop solution. Optical densities were measured at 450 nm using a microplate reader (SPECTROstar, BMG LabTech, Ortenberg, Germany). Total human SCFAs in each saliva sample was measured using an SCFA ELISA Kit (MBS7269061; MyBioSource, San Diego, CA, USA), which has a sensitivity of 0.92 pg/mL.

Plasma CPT1A levels were measured using a CPT1A ELISA Kit (MBS724213; MyBioSource) and the minimum detectable concentration was 0.1 ng/mL. Plasma IL-6 (catalog number BMS213-2; Thermo Fisher Scientific) and TNF-α (catalog number BMS223-4) levels were quantified using an ELISA kit. The minimum detectable concentrations of the kit were 0.92 pg/mL for IL-6 and 2.3 pg/mL for TNF-α.

### 4.12. Statistical Analysis

Python software (version 3.7.15) and the H2O Python module (version 3.38.0.2) (https://github.com/h2oai/h2o-3, accessed on 23 March 2024) were used for cancer diagnosis and biomarker identification. The gradient-based one-side sampling method was employed within the light gradient-boosting machine (LightGBM) model, and optimization was performed using various metrics, including accuracy, area under the receiver-operating characteristic (ROC) curve, F1 score, precision, and recall. R software (version 4.1.1) was used for analysis and visualization, with *t*-tests and chi-square tests conducted to compare observed traits. Alpha diversity was determined by observing OTUs and the Chao index, while beta diversity was assessed through principal coordinate analysis using both weighted and unweighted UniFrac analyses.

For survival analysis, we utilized the Cox proportional hazards regression model to evaluate the impact of microbial genera and other clinical factors on disease-free survival (DFS) and overall survival (OS). Both univariate and multivariate Cox analyses were performed to identify independent predictors of survival outcomes. Statistical significance across other comparisons was assessed using quartiles, Wilcoxon’s rank-sum test, fold-changes, and logistic regression. Linear discriminant analysis effect size (LEfSe) was conducted to identify genus-level microbial differences.

## 5. Conclusions

In conclusion, our findings reveal a potential association between oral microbiome dysbiosis and metabolic enzymes, such as CPT1A, alongside immunological pathways in oral cancer. Notably, shifts in bacterial composition, with higher levels of *Streptococcus* and *Parvimonas* correlating with poorer disease-free survival (DFS) and overall survival (OS), contrast with the improved survival outcomes associated with *Prevotella* and *Corynebacterium*. Our results indicate that alterations in these genera may influence critical metabolic pathways linked to cell growth, immune activation, and oxidative stress during oral cancer progression. Furthermore, the observed downregulation of SCFAs underscores the significant role of microbial metabolism in disease development. Collectively, our findings suggest that *Streptococcus*, *Parvimonas*, *Corynebacterium*, and *Prevotella* may serve as potential biomarkers for oral cancer; however, the validity of these microorganisms and metabolites as definitive biomarkers necessitates additional investigation. Continued research is crucial to confirm their roles in oral cancer and elucidate the mechanisms by which these microbial taxa impact disease progression and survival outcomes.

## Figures and Tables

**Table 1:** General characteristics of oral microbiota study subjects in discovery (n = 637), and validation (n = 385) data set.

		Discovery Data Set (n = 637)			Validation Data Set (n = 385)		
Variable		OC (N = 104)	Control (N = 533)	*p* Value	OC (N = 53)	Control (N = 332)	*p* Value
Gender	Male	67 (64.4)	254 (47.7)	0.003	29 (54.7)	105 (31.6)	0.002
	Female	37 (35.6)	279 (52.3)		24 (45.3)	227 (68.4)	
Age	I (Age < 50)	16 (15.4)	91 (17.1)	0.000	5 (9.40)	40 (12.0)	0.000
	II (Age 45–60)	28 (26.9)	241 (45.2)		5 (9.40)	142 (42.8)	
	III (Age 60–70)	19 (18.3)	168 (31.5)		17 (32.1)	140 (42.2)	
	IV (Age 70<)	41 (39.4)	33 (6.20)		26 (49.1)	10 (3.00)	
BMI	BMI < 18.5	17 (16.4)	16 (3.00)	0.000	2 (3.70)	7 (2.10)	
	BMI 18.5–23	30 (28.8)	190 (35.6)		11 (20.8)	127 (38.3)	
	BMI 23–25	19 (18.3)	137 (25.7)		16 (30.2)	90 (27.1)	0.118
	BMI ≥ 25	38 (36.5)	186 (34.9)		23 (43.4)	106 (31.9)	
	Unknown	0 (0.00)	4 (0.80)		1 (1.90)	2 (0.60)	
Smoking	Non smoker	55 (52.9)	298 (55.9)	0.010	29 (54.7)	227 (68.4)	0.206
	Currentsmoker	23 (22.1)	58 (10.9)		5 (9.40)	17 (5.10)	
	Ex-smoker	25 (24.0)	160 (30.0)		18 (34.0)	80 (24.1)	
	Unknown	1 (1.00)	17 (3.20)		1 (1.90)	8 (2.40)	
Drinking	Non drinker	42 (40.3)	119 (22.3)	0.000	35 (66.0)	97 (29.3)	<0.0001
	Currentdrinker	35 (33.7)	318 (59.7)		17 (32.1)	186 (56.0)	
	Ex-drinker	26 (25.0)	69 (12.9)		0 (0.00)	35 (10.5)	
	Unknown	1 (1.00)	27 (5.10)		1 (1.90)	14 (4.20)	
Tstage	T1	16 (15.4)		0.000	14 (26.4)		0.0017
	T2	21 (20.2)			16 (30.2)		
	T3	26 (25.0)			1 (1.90)		
	T4	34 (32.7)			16 (30.2)		
	Unknown	7 (6.70)			6 (11.3)		
Nstage	N0	62 (59.7)		0.000	36 (67.9)		<0.0001
	N1	13 (12.5)			5 (9.40)		
	N2	12 (11.5)			7 (13.2)		
	N3	10 (9.60)			1 (1.90)		
	Unknown	7 (6.70)			4 (7.60)		
Stage	1	17 (16.4)		0.000	13 (24.5)		0.002
	2	11 (10.6)			13 (24.5)		
	3	25 (24.0)			3 (5.70)		
	4	46 (44.2)			19 (35.9)		
	Unknown	5 (4.80)			5 (9.40)		
Grade	Poor	14 (13.4)		0.000	0 (0.00)		<0.001
	Moderate	43 (35.3)			4 (7.60)		
	Well	35 (30.7)			37 (69.8)		
	Unknown	12 (20.6)			12 (22.6)		

Notes: Results of variables are presented as number (%). Smoking status was categorized into three groups: non-smoker, current smoker and ex-smoker (the unknown category was used to exclude individuals with missing smoking-status information). Drinking status was also categorized into three groups: non-drinker, current drinker and ex-drinker (the unknown category was used to exclude samples with missing drinking-status information). All Stage and Grade information was collected from oral cancer patients except for those in the control group. Body mass index (BMI) was split into four groups: underweight < 18.5, normal range 18.5–23, overweight 23–25, and obese ≥ 25. Samples with missing BMI status information were excluded. OC, oral cancer.

**Table 2:** Cox proportional hazards analysis of disease-free and overall survival in oral cancer patients.

DFS	Univariate Cox Proportional			Multivariate Cox Proportional		
Microbiome	HR	95%CI	*p*-Value	HR	95%CI	*p*-Value
*Streptococcus*	2.30	(1.10–5.10)	0.035	2.85	(1.29–6.32)	0.009
*Parvimonas*	2.52	(1.26–5.42)	0.023	2.17	(0.98–4.79)	0.05
*Prevotella*	0.29	(0.26–0.91)	0.007	0.28	(0.11–0.69)	0.006
*Corynebacterium*	0.28	(0.43–1.30)	0.016	0.26	(1.35–6.03)	0.01
Overall survival						
*Streptococcus*	5.10	(1.90–13.0)	0.001	5.76	(2.17–15.3)	0.0004
*Parvimonas*	3.20	(1.40–7.30)	0.005	2.90	(1.26–6.66)	0.0119
*Prevotella*	0.29	(0.12–0.67)	0.004	0.30	(0.12–0.72)	0.0074
*Corynebacterium*	0.35	(0.14–0.87)	0.024	0.36	(0.14–0.91)	0.0318

CI, confidence interval; HR, hazard ratio; DFS, disease-free survival.

## Data Availability

The data supporting the findings of this study can be obtained from the corresponding author upon reasonable request.

## Supplementary Materials

The supporting information can be downloaded at: https://www.mdpi.com/article/10.3390/ijms252010890/s1.
