# Supplementary material for: Oral Microbiome and CPT1A Function in Fatty Acid Metabolism in Oral Cancer
Source: Int J Mol Sci. 2024 Oct 10;25(20):10890. doi: 10.3390/ijms252010890 (PMC11508181; doi:10.3390/ijms252010890)
Supplement: Supplementary file 1 [file ijms-25-10890-s001.zip › ijms-3169646-supplementary.pdf]

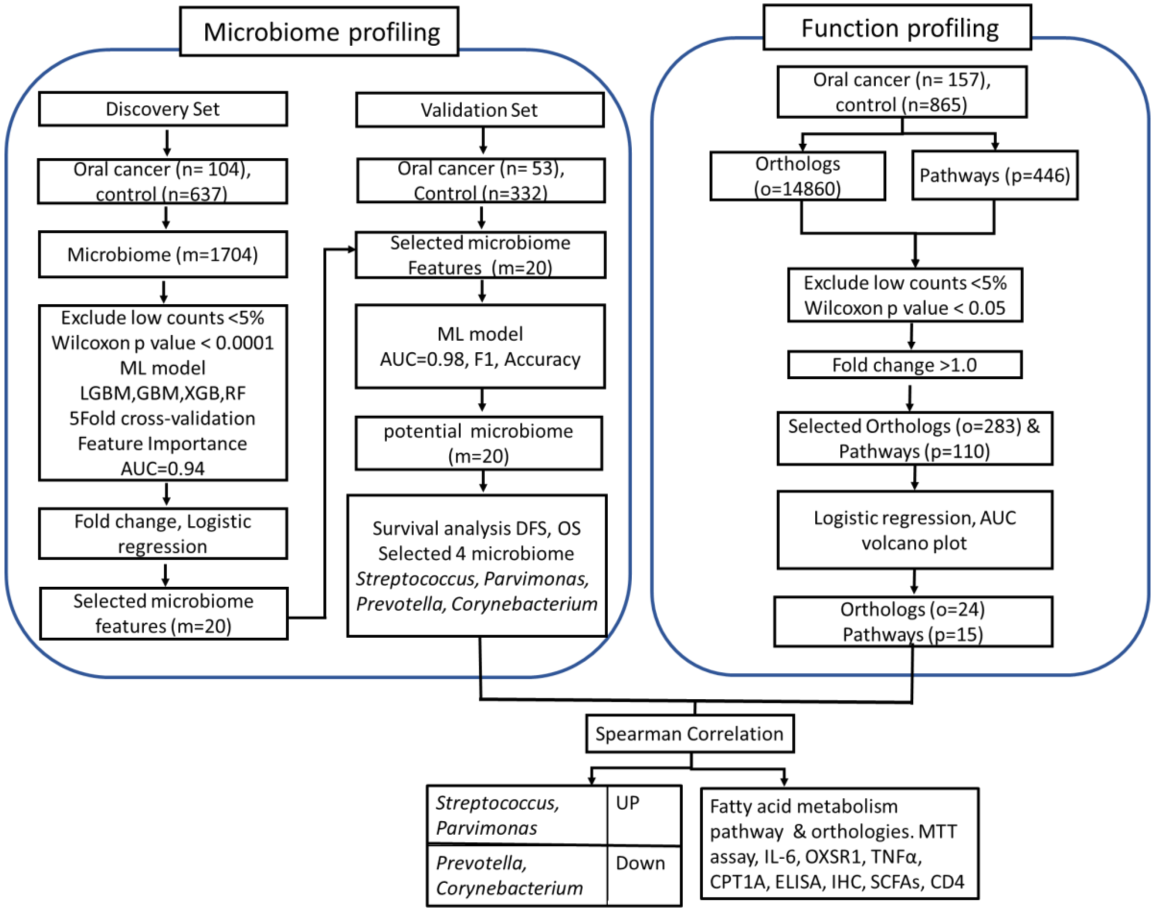

Figure S1. Utilization of Advanced Analytical Techniques in the Study: Machine Learning, Correlation, Microbiome profiling, Cell viability, ELISA, and Immunohistochemistry. HC, Healthy control; OC, Oral cancer; LGBM, light gradient-boosting machine; RF, Random Forest; XGB, Extreme Gradient Boosting; SCFAs, short chain fatty acids; OXSR1, Oxidative Stress Responsive Kinase 1; TNFα, Tumor necrosis factor alpha; CPT1A, Carnitine palmitoyltransferase 1A; IL-6, Interleukin-6; MTT assay, 3-(4, 5-dimethylthiazolyl-2)-2, 5-diphenyltetrazolium bromide, ELISA, Enzyme-linked immunosorbent test; DFS, disease-free survival; OS; overall survival.

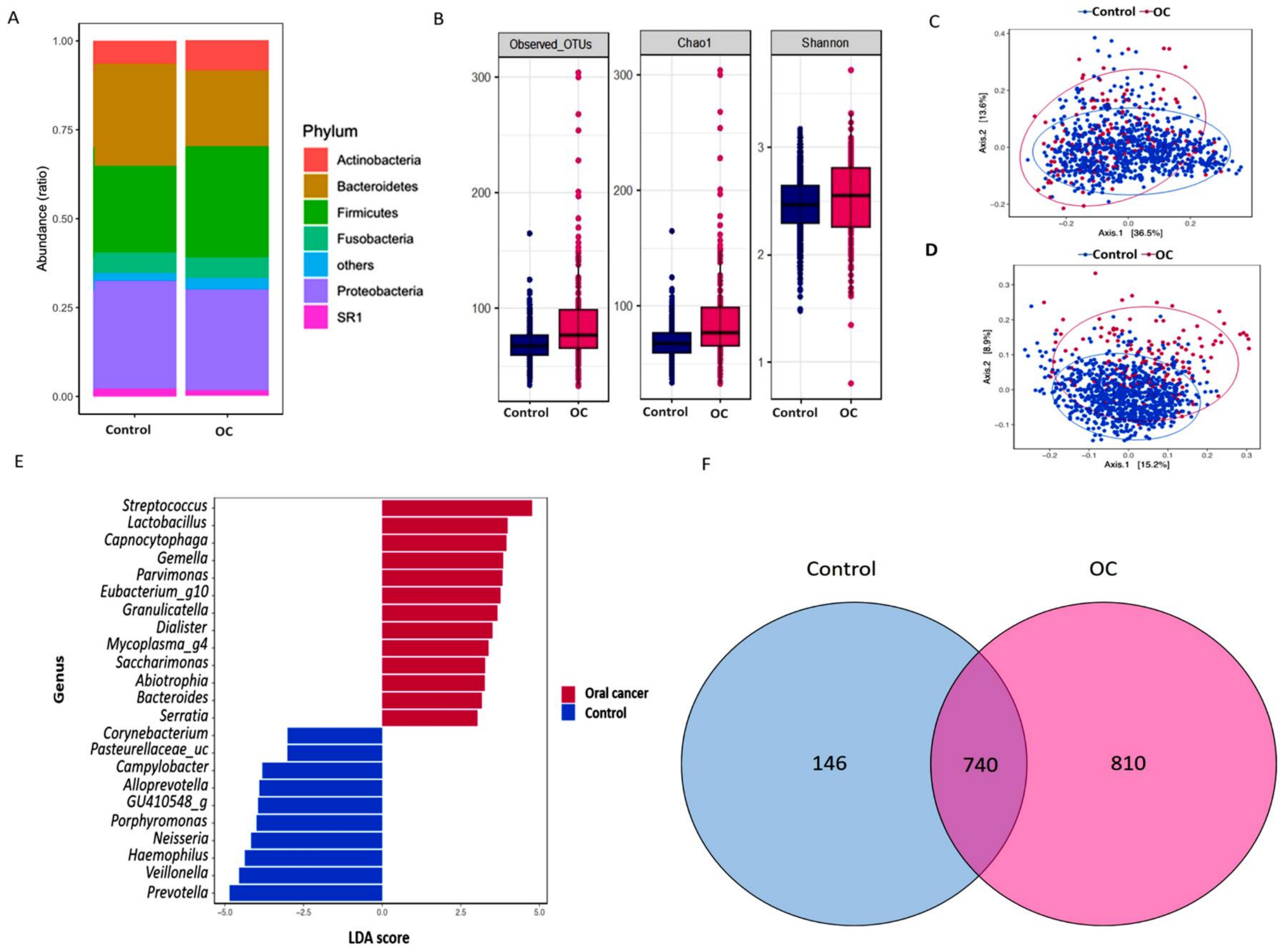

A
B
C
D
E
F
Abundance (ratio)
Control
OC
Phylum
Actinobacteria
Bacteroidetes
Firmicutes
Fusobacteria
others
Proteobacteria
SR1
Observed_OTUs
Chao1
Shannon
Axis.1 [36.5%]
Axis.2 [13.6%]
Axis.1 [15.2%]
Axis.2 [8.9%]
Streptococcus
Lactobacillus
Capnocytophaga
Gemella
Parvimonas
Eubacterium_g10
Granulicatella
Dialister
Mycoplasma_g4
Saccharimonas
Abiotrophia
Bacteroides
Serratia
Corynebacterium
Pasteurellaceae_uc
Campylobacter
Alloprevotella
GU410548_g
Porphyromonas
Neisseria
Haemophilus
Veillonella
Prevotella
Genus
LDA score
Oral cancer
Control
146
740
810

Figure S2. Diversity analysis of oral microbiota in oral cancer and control group. (A) Average composition of bacterial community at phylum levels with the relative abundance greater than 1%. (B) Alpha diversity was estimated by observing operational taxonomic units (OTUs, $P<0.001$), Chao and the Shannon index ($P<0.001$). (C) Beta diversity was calculated using principal coordinate analysis based on weighted ($P<0.001$) (D) unweighted ($P<0.001$) UniFrac distances in oral microbiota communities. (E) LEfSe analysis Linear Discriminant Analysis (LDA) at the genus level in oral cancer and control groups. **(F)** Venn diagrams showing overlaps between groups at the genus level in oral cancer and control groups. OC, oral cancer

Fold 1

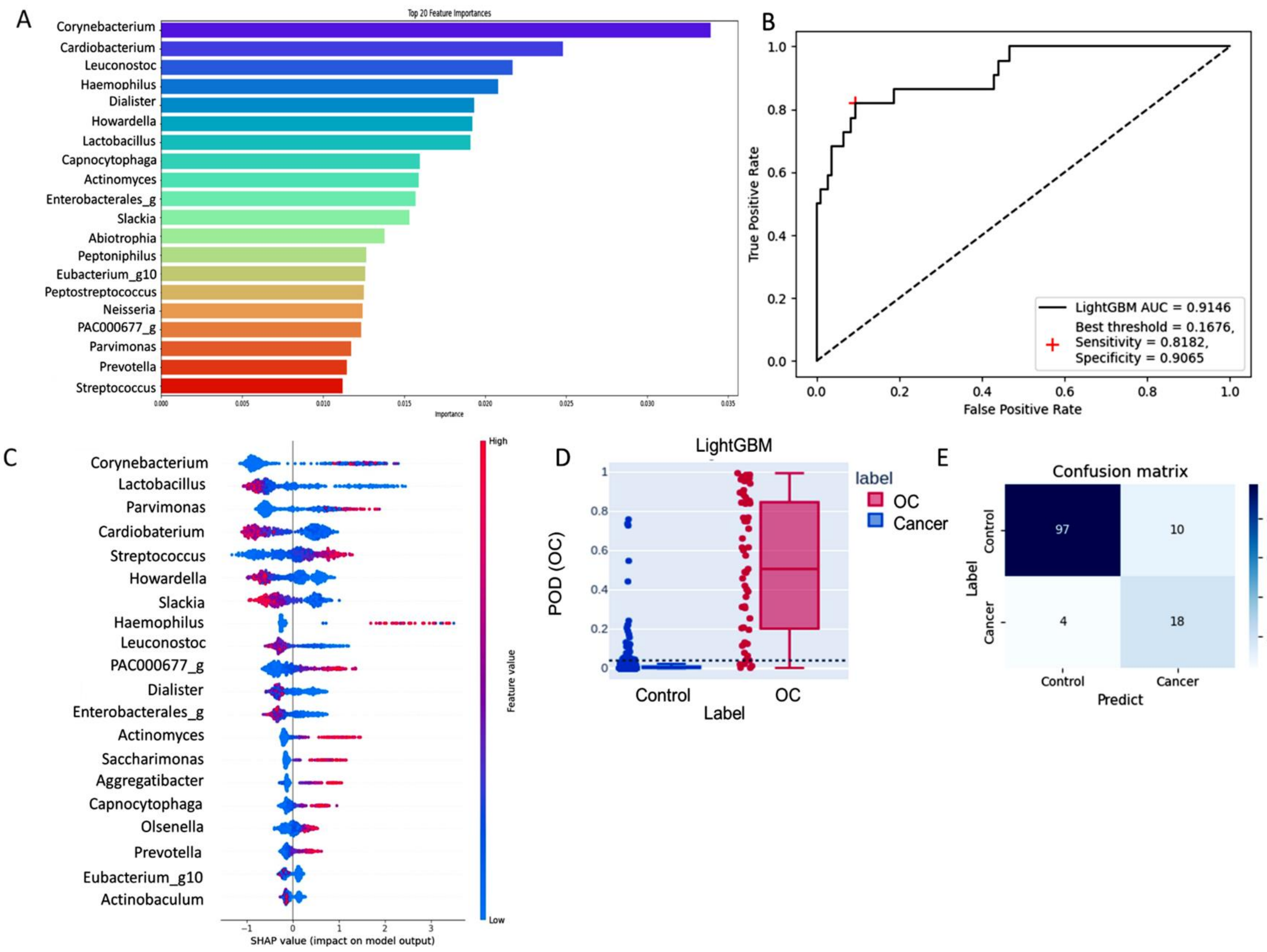

A
Top 20 Feature Importances
Corynebacterium
Cardiobacterium
Leuconostoc
Haemophilus
Dialister
Howardella
Lactobacillus
Capnocytophaga
Actinomyces
Enterobacterales_g
Slackia
Abiotrophia
Peptoniphilus
Eubacterium_g10
Peptostreptococcus
Neisseria
PAC000677_g
Parvimonas
Prevotella
Streptococcus
Importance
B
True Positive Rate
False Positive Rate
LightGBM AUC = 0.9146
Best threshold = 0.1676,
Sensitivity = 0.8182,
Specificity = 0.9065
C
Corynebacterium
Lactobacillus
Parvimonas
Cardiobaterium
Streptococcus
Howardella
Slackia
Haemophilus
Leuconostoc
PAC000677_g
Dialister
Enterobacterales_g
Actinomyces
Saccharimonas
Aggregatibacter
Capnocytophaga
Olsenella
Prevotella
Eubacterium_g10
Actinobaculum
SHAP value (impact on model output)
High
Feature value
Low
D
LightGBM
POD (OC)
label
OC
Cancer
Control
OC
Label
E
Confusion matrix
Label
Control
Cancer
97
10
4
18
Predict

Fold 2

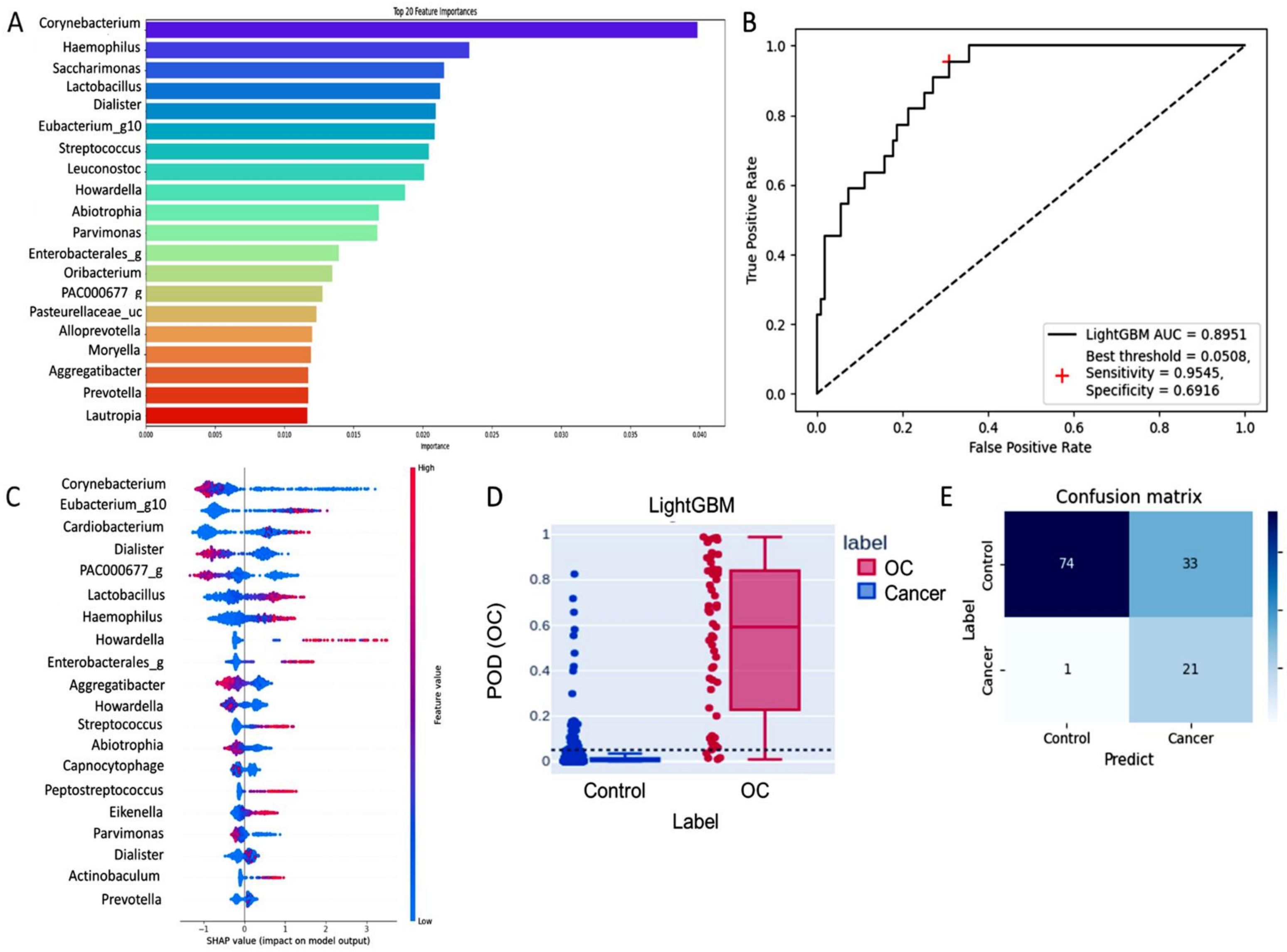
A
Top 20 Feature Importances
Corynebacterium
Haemophilus
Saccharimonas
Lactobacillus
Dialister
Eubacterium_g10
Streptococcus
Leuconostoc
Howardella
Abiotrophia
Parvimonas
Enterobacterales_g
Oribacterium
PAC000677_g
Pasteurellaceae_uc
Alloprevotella
Moryella
Aggregatibacter
Prevotella
Lautropia
Importance
B
True Positive Rate
False Positive Rate
LightGBM AUC = 0.8951
Best threshold = 0.0508,
Sensitivity = 0.9545,
Specificity = 0.6916
C
Corynebacterium
Eubacterium_g10
Cardiobacterium
Dialister
PAC000677_g
Lactobacillus
Haemophilus
Howardella
Enterobacterales_g
Aggregatibacter
Howardella
Streptococcus
Abiotrophia
Capnocytophage
Peptostreptococcus
Eikenella
Parvimonas
Dialister
Actinobaculum
Prevotella
SHAP value (impact on model output)
High
Feature value
Low
D
LightGBM
POD (OC)
label
OC
Cancer
Control
OC
Label
E
Confusion matrix
Label
Control
Cancer
74
33
1
21
Control
Cancer
Predict

Fold 3

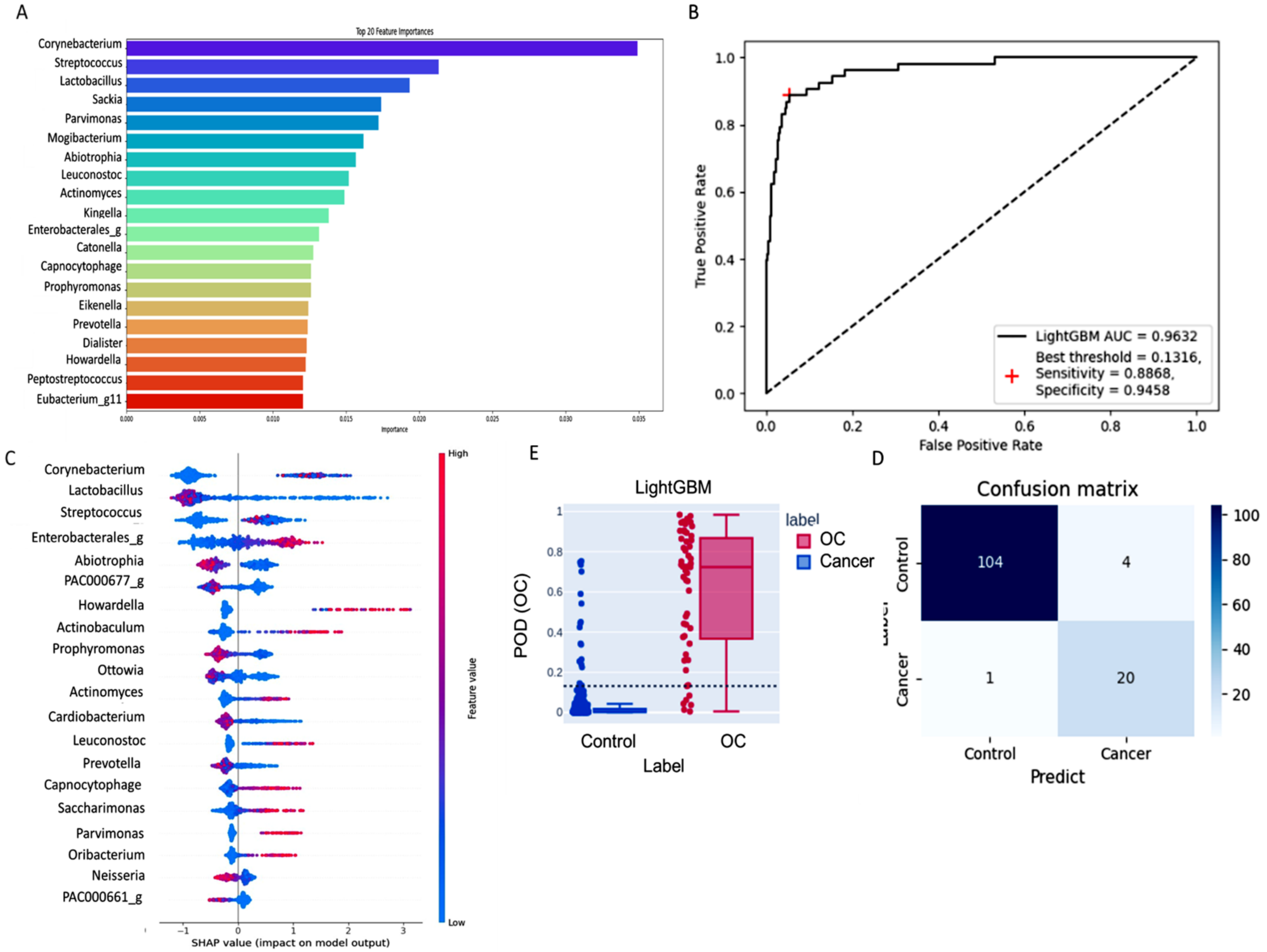

A
Top 20 Feature Importances
Corynebacterium
Streptococcus
Lactobacillus
Sackia
Parvimonas
Mogibacterium
Abiotrophia
Leuconostoc
Actinomyces
Kingella
Enterobacterales_g
Catonella
Capnocytophage
Prophyromonas
Eikenella
Prevotella
Dialister
Howardella
Peptostreptococcus
Eubacterium_g11
B
True Positive Rate
False Positive Rate
LightGBM AUC = 0.9632
Best threshold = 0.1316,
Sensitivity = 0.8868,
Specificity = 0.9458
C
Corynebacterium
Lactobacillus
Streptococcus
Enterobacterales_g
Abiotrophia
PAC000677_g
Howardella
Actinobaculum
Prophyromonas
Ottowia
Actinomyces
Cardiobacterium
Leuconostoc
Prevotella
Capnocytophage
Saccharimonas
Parvimonas
Oribacterium
Neisseria
PAC000661_g
SHAP value (impact on model output)
High
Low
Feature value
E
LightGBM
POD (OC)
label
OC
Cancer
Control
OC
Label
D
Confusion matrix
Control
Cancer
104
4
1
20
Control
Cancer
Predict

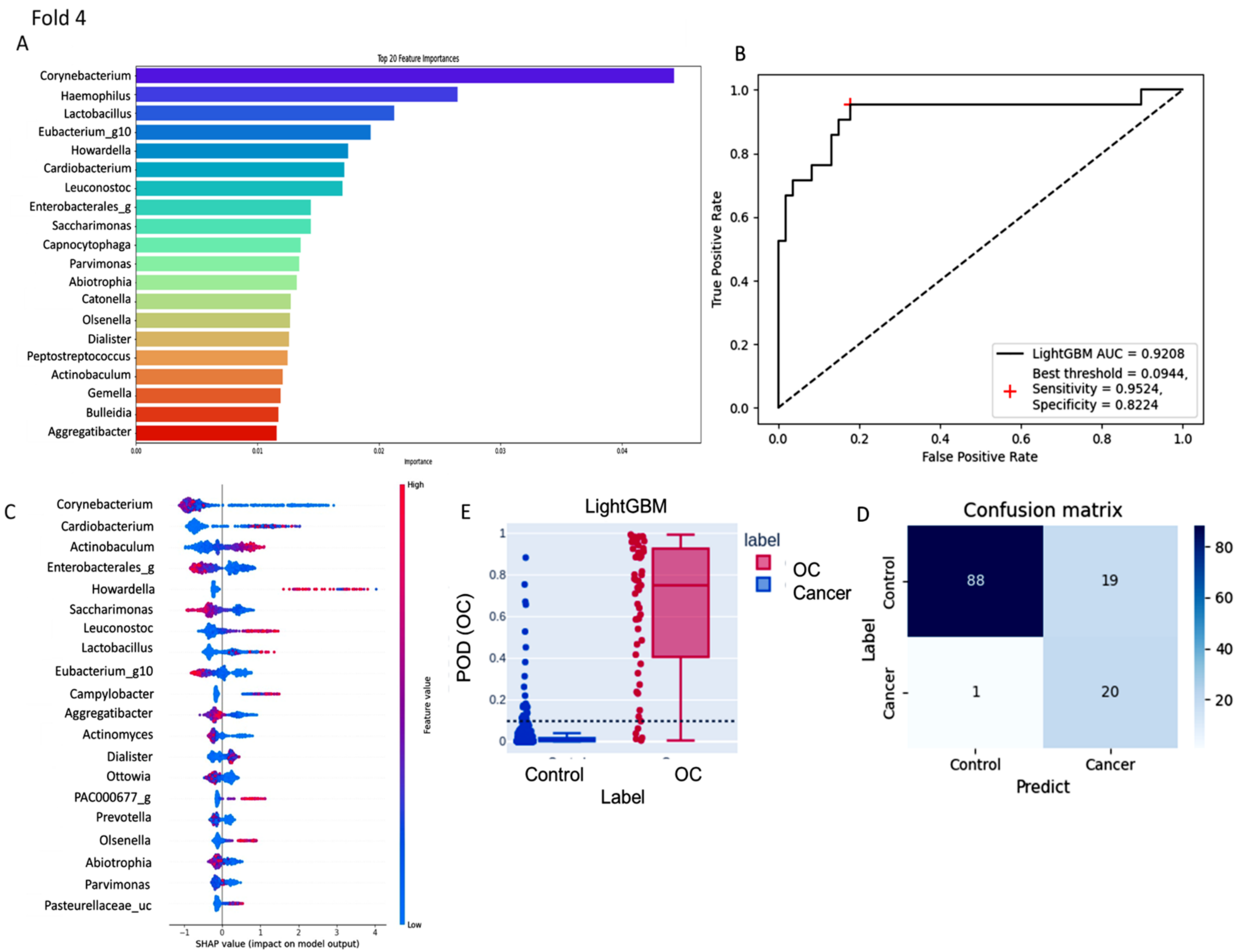

Fold 4
A
Top 20 Feature Importances
Corynebacterium
Haemophilus
Lactobacillus
Eubacterium_g10
Howardella
Cardiobacterium
Leuconostoc
Enterobacterales_g
Saccharimonas
Capnocytophaga
Parvimonas
Abiotrophia
Catonella
Olsenella
Dialister
Peptostreptococcus
Actinobaculum
Gemella
Bulleidia
Aggregatibacter
Importance
B
True Positive Rate
False Positive Rate
LightGBM AUC = 0.9208
Best threshold = 0.0944,
Sensitivity = 0.9524,
Specificity = 0.8224
C
Corynebacterium
Cardiobacterium
Actinobaculum
Enterobacterales_g
Howardella
Saccharimonas
Leuconostoc
Lactobacillus
Eubacterium_g10
Campylobacter
Aggregatibacter
Actinomyces
Dialister
Ottowia
PAC000677_g
Prevotella
Olsenella
Abiotrophia
Parvimonas
Pasteurellaceae_uc
SHAP value (impact on model output)
Feature value
High
Low
E
LightGBM
POD (OC)
label
OC
Cancer
Control
OC
Label
D
Confusion matrix
88
19
1
20
Control
Cancer
Label
Predict

Fold 5

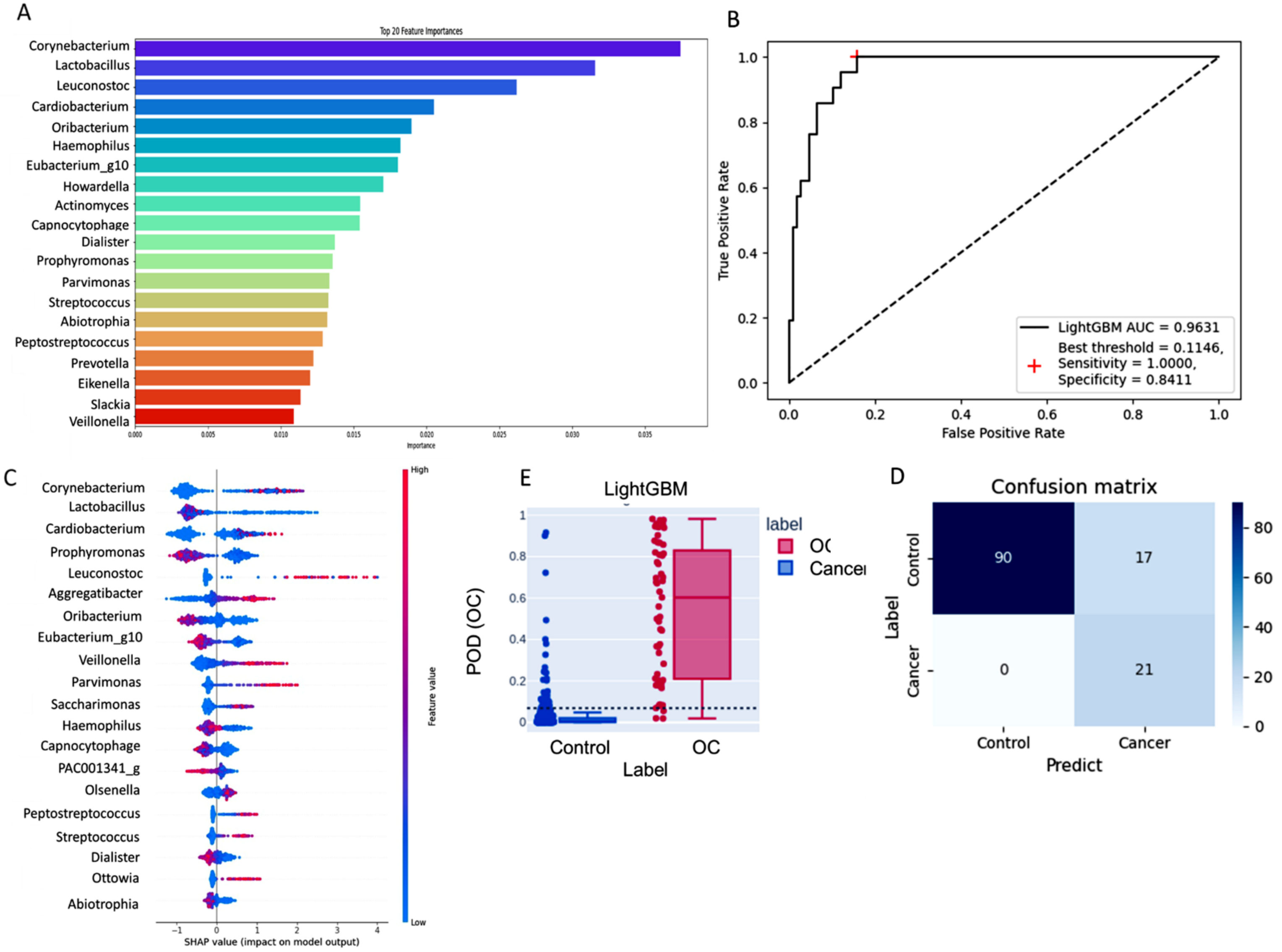
A
Top 20 Feature Importances
Corynebacterium
Lactobacillus
Leuconostoc
Cardiobacterium
Oribacterium
Haemophilus
Eubacterium_g10
Howardella
Actinomyces
Capnocytophage
Dialister
Prophyromonas
Parvimonas
Streptococcus
Abiotrophia
Peptostreptococcus
Prevotella
Eikenella
Slackia
Veillonella
Importance
B
True Positive Rate
False Positive Rate
LightGBM AUC = 0.9631
Best threshold = 0.1146,
Sensitivity = 1.0000,
Specificity = 0.8411
C
Corynebacterium
Lactobacillus
Cardiobacterium
Prophyromonas
Leuconostoc
Aggregatibacter
Oribacterium
Eubacterium_g10
Veillonella
Parvimonas
Saccharimonas
Haemophilus
Capnocytophage
PAC001341_g
Olsenella
Peptostreptococcus
Streptococcus
Dialister
Ottowia
Abiotrophia
SHAP value (impact on model output)
High
Feature value
Low
E
LightGBM
POD (OC)
Control
OC
Label
label
OC
Cancer
D
Confusion matrix
Label
Control
Cancer
90
17
0
21
Control
Cancer
Predict

Figure S3. 5Fold ML result prediction of oral cancer using LightGBM model. Fold1, Fold2, Fold3, Fold4, Fold5 Feature importance, ROC curve, Confusion matrix, SHAP value and POD. SHAP, SHapley Additive exPlanations; ROC curve, Receiver Operating Characteristic curve; POD, Probability of Detection Index; LightGBM, Light Gradient Boosting Machine learning model.

Table S1. Metrics based on 4 machine learning techniques.

	LGBM	GBM	RF	XGB
AUC score	0.9857	0.8452	0.9655	0.9629
Accuracy	0.9767	0.8873	0.9429	0.9429
Specificity	0.9940	0.9247	0.9831	0.9729
Sensitivity	0.9636	0.6528	0.6906	0.7547
Precision	0.9818	0.5808	0.8670	0.8170
F1 score	0.906	0.6140	0.7685	0.7832

LGBM, light gradient boosted machine; GBM, gradient boosted machine; RF, Random Forest; XGB, extreme gradient boosting.

Table S2. Distribution and fold change of 20 microbiota in microbiome data set.

Feature	FC	log2FC	*p*-value [a]	-LOG10(*p*)
Streptococcus	1.964	0.974	4.85E-15	14.314
Lactobacillus	14.71	3.879	4.31E-14	13.365
Parvimonas	1.951	0.964	5.08E-10	9.2940
Prevotella	0.590	-0.761	4.48E-09	8.3484
Capnocytophaga	2.828	1.500	3.95E-07	6.4035
Abiotrophia	11.97	3.582	4.47E-07	6.3496
Alloprevotella	0.466	-1.102	3.18E-06	5.4972
Campylobacter	0.601	-0.734	1.44E-05	4.8410
Enterobacter	53.34	5.737	3.09E-04	3.5103
Rhodococcus	18.88	4.239	5.51E-04	3.2587
Actinobaculum	0.372	-1.428	2.76E-03	2.5594
Dialister	1.540	0.623	3.58E-03	2.4464
Haemophilus	0.787	-0.345	4.76E-03	2.3225
Corynebacterium	0.543	-0.882	5.81E-03	2.2360
PAC000677	0.427	-1.228	9.83E-03	2.0075
Veillonella	0.833	-0.264	3.08E-02	1.5112
Porphyromonas	0.788	-0.343	5.81E-02	1.2359
Howardella	0.367	-1.444	2.09E-01	0.6798
Eubacterium	0.805	-0.313	2.52E-01	0.5987
Aggregatibacter	0.796	-0.330	2.58E-01	0.5892

20 Microbiota were shown as fold change according to oral cancer and control groups. [a]*p*-value obtained as a result of wilcoxon rank-sum test of continuous variable.

Table S3. Logistic regression analysis of 20 genera for oral cancer risks in the LightGBM model.

Microbiome	Logistic Regression	OC	Control	Univariate OR (95%CI)	Univariate *p*[b]	Multivariate OR[a] (95%CI)	Multivariate *p*[b]
Streptococcus	Continuous scale	157	865	1.10 (1.08~1.13)	2.01E-16	1.06 (1.03~1.09)	2.46E-05
	Quartile4(>7.06)	101	216	5.97 (3.45~10.3)	3.14E-10	3.52 (1.80~6.88)	5.18E-04
	Quartile3(4.51-7.06)	24	216	1.42 (0.74~2.71)	3.89E-01	1.08 (0.50~2.34)	8.43E-01
	Quartile2(2.81-4.51)	15	216	0.89 (0.43~1.82)	7.43E-01	0.73 (0.31~1.68)	5.83E-01
	Quartile1(<2.81)	17	217	ref		ref	
Parvimonas	Continuous scale	157	865	6.84 (4.18~11.1)	2.01E-14	5.44 (2.95~10.0)	9.62E-08
	Quartile4(>0.17)	80	216	3.21 (1.98~5.23)	5.21E-06	2.64 (1.41~4.93)	5.16E-03
	Quartile3(0.07-0.17)	22	216	0.88 (0.48~1.62)	6.89E-01	0.75 (0.35~1.61)	5.91E-01
	Quartile2(0.02-0.07)	30	216	1.21 (0.69~2.12)	6.87E-01	1.21 (0.59~2.45)	6.06E-01
	Quartile1(<0.02)	25	217	ref		ref	
Corynebacterium	Continuous scale	157	865	0.33 (0.16~0.69)	2.91E-03	0.38 (0.20~0.72)	4.89E-03
	Quartile4(>0.39)	23	216	0.22 (0.14~0.36)	1.69E-09	0.20 (0.11~0.38)	1.25E-06
	Quartile3(0.20-0.39)	15	216	0.14 (0.08~0.26)	1.06E-10	0.17 (0.08~0.34)	1.25E-06
	Quartile2(0.09-0.20)	15	216	0.14 (0.08~0.26)	1.06E-10	0.11 (0.05~0.24)	4.86E-08
	Quartile1(<0.09)	104	217	ref		ref	
Prevotella	Continuous scale	157	865	0.93 (0.92~0.95)	3.86E-11	0.94 (0.92~0.96)	1.65E-07
	Quartile4(>26.99)	15	216	0.19 (0.11~0.34)	5.18E-08	0.17 (0.08~0.35)	3.66E-06
	Quartile3(17.26-26.99)	24	216	0.31 (0.19~0.50)	3.35E-06	0.29 (0.15~0.56)	3.71E-04
	Quartile2(10.45-17.26)	39	216	0.50 (0.32~0.76)	1.30E-03	0.49 (0.28~0.88)	2.05E-02
	Quartile1(<10.45)	79	217	ref		ref	
Actinobaculum	Continuous scale	157	865	1.22 (1.00~1.50)	5.46E-02	1.41 (1.08~1.86)	1.78E-02
	Quartile4(>0.04)	13	216	0.19 (0.11~0.34)	2.58E-08	0.24 (0.12~0.49)	1.37E-04
	Quartile3(0.01-0.04)	26	216	0.30 (0.19~0.49)	1.84E-06	0.36 (0.19~0.68)	3.26E-03
	Quartile2(0.00-0.01)	32	215	0.39 (0.25~0.61)	3.53E-05	0.43 (0.24~0.77)	6.01E-03

	Quartile1(<0.00)	86	218	ref		ref	
PAC000677	Continuous scale	157	865	0.55 (0.05~6.55)	6.34E-01	0.14 (0.01~2.79)	2.80E-01
	Quartile4(>0.04)	23	216	0.27 (0.17~0.45)	4.65E-07	0.25 (0.13~0.50)	1.26E-04
	Quartile3(0.02-0.04)	17	215	0.20 (0.12~0.35)	3.56E-08	0.24 (0.12~0.49)	1.41E-04
	Quartile2(0.00-0.02)	32	215	0.38 (0.25~0.60)	2.74E-05	0.50 (0.28~0.89)	2.39E-02
	Quartile1(<0.00)	85	219	ref		ref	
Aggregatibacter	Continuous scale	157	865	0.96 (0.79~1.15)	6.25E-01	1.01 (0.84~1.21)	9.39E-01
	Quartile4(>0.79)	20	216	0.25 (0.15~0.42)	3.76E-07	0.36 (0.19~0.70)	4.93E-03
	Quartile3(0.28-0.79)	28	216	0.35 (0.22~0.56)	9.94E-06	0.61 (0.34~1.10)	1.29E-01
	Quartile2(0.06-0.28)	28	216	0.35 (0.22~0.56)	9.94E-06	0.49 (0.26~0.91)	3.60E-02
	Quartile1(<0.06)	81	217	ref		ref	
Eubacterium	Continuous scale	157	865	0.36 (0.09~1.37)	1.34E-01	0.54 (0.13~2.31)	4.10E-01
	Quartile4(>0.04)	32	216	0.38 (0.24~0.60)	2.72E-05	0.41 (0.23~0.75)	5.05E-03
	Quartile3(0.02-0.04)	22	216	0.26 (0.16~0.44)	3.06E-07	0.28 (0.15~0.54)	3.32E-04
	Quartile2(0.00-0.02)	19	216	0.23 (0.13~0.39)	9.77E-08	0.31 (0.16~0.60)	1.02E-03
	Quartile1(<0.00)	84	217	ref		ref	
Alloprevotella	Continuous scale	157	865	0.85 (0.79~0.92)	4.17E-05	0.96 (0.89~1.03)	3.65E-01
	Quartile4(>0.79)	21	216	0.22 (0.13~0.36)	5.26E-09	0.42 (0.23~0.78)	8.69E-03
	Quartile3(0.28-0.79)	17	216	0.18 (0.10~0.30)	1.10E-09	0.30 (0.15~0.59)	9.14E-04
	Quartile2(0.06-0.28)	22	216	0.23 (0.14~0.38)	6.58E-09	0.27 (0.14~0.50)	9.93E-05
	Quartile1(<0.06)	97	217	ref		ref	
Porphyromonas	Continuous scale	157	865	0.94 (0.89~0.98)	7.86E-03	0.97 (0.85~1.09)	3.45E-01
	Quartile4(>6.48)	28	216	0.41 (0.26~0.67)	4.02E-04	0.71 (0.38~1.33)	4.24E-01
	Quartile3(3.48-6.48)	24	216	0.35 (0.21~0.59)	1.03E-04	0.33 (0.17~0.65)	3.12E-03
	Quartile2(1.51-3.48)	37	216	0.55 (0.35~0.85)	7.47E-03	0.98 (0.55~1.73)	9.40E-01
	Quartile1(<1.51)	68	217	ref		ref	
Haemophilus	Continuous scale	157	865	0.92 (0.90~0.95)	8.74E-08	0.98 (0.95~1.01)	3.16E-01
	Quartile4(>15.57)	28	216	0.30 (0.19~0.47)	2.38E-07	0.68 (0.37~1.23)	2.61E-01

	Quartile3(10.88-15.57)	17	216	0.18 (0.10~0.31)	1.24E-09	0.32 (0.17~0.61)	9.97E-04
	Quartile2(6.85-10.88)	17	216	0.18 (0.10~0.31)	1.86E-09	0.27 (0.13~0.54)	4.89E-04
	Quartile1(<6.85)	95	217	ref		ref	
Veillonella	Continuous scale	157	865	0.94 (0.92~0.97)	7.15E-06	0.88 (0.80~0.96)	4.20E-06
	Quartile4(>17.72)	19	216	0.28 (0.17~0.49)	1.17E-05	0.15 (0.07~0.31)	8.83E-07
	Quartile3(11.99-17.72)	29	216	0.43 (0.27~0.70)	7.79E-04	0.44 (0.23~0.83)	1.64E-02
	Quartile2(7.34-11.99)	42	216	0.63 (0.41~0.97)	3.48E-02	0.59 (0.33~1.05)	9.16E-02
	Quartile1(<7.34)	67	217	ref		ref	
Campylobacter	Continuous scale	157	865	0.64 (0.53~0.78)	7.29E-06	0.63 (0.50~0.80)	1.87E-04
	Quartile4(>2.11)	20	216	0.31 (0.18~0.53)	3.45E-05	0.26 (0.13~0.52)	2.91E-04
	Quartile3(1.27-2.11)	34	216	0.53 (0.33~0.83)	7.52E-03	0.45 (0.25~0.82)	1.38E-02
	Quartile2(0.70-1.27)	38	216	0.59 (0.38~0.91)	1.84E-02	0.57 (0.31~1.03)	7.94E-02
	Quartile1(<0.70)	65	217	ref		ref	
Howardella	Continuous scale	157	865	inf	7.29E-06	inf	4.69E-04
	>0	83	154	5.18 (3.62~7.41)	3.38E-12	3.78 (2.35~6.10)	8.29E-08
	,0	74	711	ref	2.47E-19	ref	
Capnocytophaga	Continuous scale	157	865	1.30 (1.18~1.43)	5.68E-08	1.27 (1.13~1.42)	9.59E-05
	Quartile4(>1.47)	68	216	2.07 (1.31~3.27)	3.57E-03	2.49 (1.34~4.61)	8.46E-03
	Quartile3(0.81-1.47)	26	216	0.79 (0.46~1.37)	5.37E-01	1.25 (0.62~2.51)	5.32E-01
	Quartile2(0.39-0.81)	30	216	0.91 (0.54~1.55)	7.37E-01	1.31 (0.66~2.60)	4.90E-01
	Quartile1(<0.39)	33	217	ref		ref	
Dialister	Continuous scale	157	865	5.69 (3.45~9.39)	1.02E-11	3.75 (1.99~7.06)	7.30E-05
	Quartile4(>0.21)	72	216	2.70 (1.67~4.37)	9.97E-05	2.21 (1.19~4.10)	2.16E-02
	Quartile3(0.10-0.21)	30	216	1.13 (0.65~1.96)	8.35E-01	1.05 (0.51~2.17)	9.10E-01
	Quartile2(0.04-0.10)	28	214	1.06 (0.61~1.86)	8.35E-01	1.04 (0.51~2.12)	9.10E-01
	Quartile1(<0.04)	27	219	ref		ref	
Abiotrophia	Continuous scale	157	865	1.05 (0.94~1.17)	2.65E-08	29.2(5.2~163.1)	2.10E-04

	Quartile4(>0.11)	65	216	2.11 (1.42~3.14)	2.89E-05	2.77 (1.62~4.73)	4.24E-04
	Quartile3(0.06-0.11)	29	214	0.95 (0.59~1.54)	8.36E-01	1.06 (0.57~1.98)	8.58E-01
	Quartile2(0.02-0.06)	8	49	1.15 (0.52~2.55)	1.73E-01	1.49 (0.40~5.56)	6.20E-01
	Quartile1(<0.02)	55	386	ref		ref	
Enterobacter	Continuous scale	157	865	1.46 (0.97~2.19)	6.86E-02	1.15 (0.77~1.73)	4.94E-01
	>0	105	244	5.14 (3.57~7.39)	1.17E-18	4.66 (2.89~7.50)	5.85E-10
	,0	52	621	ref		ref	
Lactobacillus	Continuous scale	157	865	132.5(27.3~642.1)	1.28E-09	21.6 (4.75~99.07)	1.26E-04
	>0	140	373	10.86 (6.45~18.29)	2.91E-19	6.25 (3.49~11.20)	1.69E-09
	,0	17	492	ref		ref	
Rhodococcus	Continuous scale	157	865	inf	6.89E-09	inf	1.12E-02
	>0	18	7	15.87 (6.51~38.70)	8.52E-07	8.84 (2.28~34.28)	2.86E-03
	,0	139	858	ref	3.08E-05	ref	4.89E-03

Logistic regression analysis was performed with log scaled data. [a]Multivariate logistic regression was adjusted for sex, age, smoking, drinking and BMI status. [b]*p* value represents significance of the regression coefficient. OR, Odds ratio; LR, Logistic regression; OC, oral cancer; CI, confidence interval.

Table S4**.** Distribution and fold change of 24 orthologs in function data set.

**Orthologs**	**FC**	**log2FC**	***p*-value[a]**	**-LOG10(*p*)**
Carnitine O-palmitoyltransferase 1(K08765)	761.8	9.573	2.10E-22	21.68
Acyl-CoA dehydrogenase (K06445)	12.80	3.678	1.40E-07	6.852
long-chain acyl-CoA synthetase (K01897)	9.866	3.302	3.06E-25	24.51
H+-transporting ATPase (K01535)	148.8	7.217	8.12E-23	22.09
Diacylglycerol choline phosphotransferase (K00994)	222.9	7.800	7.62E-33	32.12
Dihydrofolate reductase (K18590)	0.030	-5.043	2.77E-21	20.56
Solute carrier family 39 member 11 (K14717)	0.031	-5.009	2.12E-08	7.673
Tropomyosin (K17945)	0.854	-0.228	3.34E-16	15.48
Kinesin family member 14 (K17915)	1.300	0.379	1.11E-10	9.956
Nonribosomal peptide synthetase protein(K16112)	2.972	1.571	2.42E-09	8.615
Carbonic anhydrase 2 (K18245)	10.47	3.388	6.77E-12	11.17
Solute carrier family 10 (K14342)	0.088	-3.510	2.27E-13	12.64
2-hydroxyacyl-CoA lyase (K12261)	1.003	0.004	8.43E-10	9.074
GDP-perosamine N-acetyltransferase (K17939)	1.627	0.702	4.14E-05	4.383
Son of sevenless (K03099)	0.741	-0.433	6.74E-06	5.172
Benzoxazolinate moiety biosynthesis protein (K21176)	1.003	0.004	8.43E-10	9.074
Enoyl-[acyl-carrier protein] reductase (K00209)	2.513	1.330	6.92E-13	12.16
Heterogeneous nuclear ribonucleoprotein (K15047)	11.33	3.502	1.98E-01	0.702
Fatty acid synthase subunit alpha (K00667)	281.4	8.137	1.12E-16	15.95
Succinylornithine aminotransferase (K00840)	190.0	7.570	3.52E-16	15.45
Alpha-D-ribose 1-methylphosphonate (K06164)	273.6	8.096	1.51E-16	15.82
Aryl-alcohol dehydrogenase (K00055)	33.90	5.083	3.98E-31	30.40
Acetyl-CoA acyltransferase (K07509)	0.164	-2.610	5.24E-04	3.281
Long-chain-fatty-acid-CoA ligase (K15013)	0.596	-0.746	1.04E-19	18.98

24 Orthologs were shown as fold change according to oral cancer and control groups. [a] *p*-value obtained as a result of wilcoxon rank-sum test of continuous variable.

Table S5. Logistic regression analysis of 24 orthologs for oral cancer risk.

Function	Logistic Regression	OC	Control	Univariate OR (95%CI)	Univariate p[b]	Multivariate OR[a] (95%CI)	Multivariate p[b]
Carnitine O-palmitoyltransferase 1(K08765)	Continuous scale	157	865	1.32 (1.11~1.57)	1.76E-03	1.49 (1.19~1.86)	7.60E-04
	Quartile4(>0.14)	36	215	2.01 (1.18~3.43)	1.41E-02	2.84 (1.44~5.62)	3.91E-03
	Quartile3(-0.27-0.14)	16	217	2.14 (1.26~3.64)	9.79E-03	3.15 (1.60~6.18)	1.93E-03
	Quartile2(-0.50-0.27)	20	214	1.70 (0.98~2.95)	5.70E-02	2.37 (1.20~4.68)	1.69E-02
	Quartile1(<-0.50)	85	219	ref		ref	
H+-transporting ATPase (K01535)	Continuous scale	157	865	10.2 (9.99~12.7)	6.19E-14	15.82 (10.5~19.3)	2.67E-04
	Quartile4(>-0.06)	105	216	13.1 (6.27~27.7)	2.06E-11	7.39 (3.30~16.54)	2.56E-06
	Quartile3(-0.07-0.06)	26	214	3.30 (1.46~7.44)	5.48E-03	2.85 (1.18~6.93)	3.08E-02
	Quartile2(-0.07-0.07)	18	218	2.24 (0.95~5.26)	6.42E-02	2.75 (1.10~6.87)	3.93E-02
	Quartile1(<-0.07)	8	217	ref		ref	
Diacylglycerol cholinephosphotransferase (K00994)	Continuous scale	157	865	1.19 (1.01~1.42)	3.97E-02	1.31 (1.06~1.62)	1.86E-02
	Quartile4(>0.68)	46	216	1.71 (1.03~2.85)	5.24E-02	2.32 (1.21~4.46)	1.69E-02
	Quartile3(-0.07-0.68)	47	216	1.75 (1.05~2.91)	6.30E-02	2.50 (1.31~4.79)	1.00E-02
	Quartile2(-0.77-0.07)	37	216	1.38 (0.81~2.34)	2.38E-01	1.95 (1.01~3.78)	6.18E-02
	Quartile1(<-0.77)	27	217	ref		ref	
long-chain acyl-CoA synthetase (K01897)	Continuous scale	157	865	0.44 (0.36~0.54)	4.39E-15	0.51 (0.40~0.64)	1.35E-08
	Quartile4(>0.64)	23	216	0.25 (0.15~0.41)	5.69E-08	0.25 (0.14~0.47)	2.17E-05
	Quartile3(0.01-0.64)	15	216	0.16 (0.09~0.29)	1.64E-09	0.15 (0.07~0.30)	2.14E-07
	Quartile2(-0.50-0.01)	27	216	0.29 (0.18~0.47)	3.24E-07	0.30 (0.16~0.55)	1.28E-04
	Quartile1(<-0.50)	92	217	ref		ref	
Acyl-CoA dehydrogenase (K06445)	Continuous scale	157	865	1.96 (1.40~2.74)	9.61E-05	1.97 (1.19~3.25)	1.17E-02
	Quartile4(>-0.09)	48	216	3.01 (1.66~5.47)	3.84E-04	2.11 (1.04~4.27)	5.81E-02
	Quartile3(-0.18-0.09)	49	216	3.08 (1.70~5.58)	4.27E-04	2.36 (1.17~4.75)	2.95E-02
	Quartile2(-0.20-0.18)	44	216	2.76 (1.51~5.05)	9.45E-04	1.77 (0.87~3.62)	1.48E-01
	Quartile1(<-0.20)	16	217	ref		ref	
Aryl-alcohol dehydrogenase (K00055)	Continuous scale	157	865	2.31 (0.91~13.1)	3.46E-01	2.47 (0.93~4.21)	6.42E-01
	Quartile4(>0.23)	26	216	0.35 (0.23~0.54)	1.86E-06	0.49 (0.28~0.85)	1.43E-02

	Quartile3(-0.26-0.23)	16	216	0.16 (0.09~0.29)	3.60E-10	0.20 (0.11~0.39)	3.12E-06
	Quartile2(-0.52-0.26)	7	216	0.09 (0.05~0.19)	1.69E-10	0.14 (0.06~0.30)	1.70E-06
	Quartile1(<-0.52)	108	217	ref		ref	
Benzoxazolinate moiety biosynthesis protein (K21176)	Continuous scale	157	865	0.77 (0.60~0.97)	2.91E-02	0.92 (0.75~1.14)	4.73E-01
	Quartile4(>0.23)	26	216	0.24 (0.15~0.39)	3.12E-09	0.35 (0.19~0.63)	6.39E-04
	Quartile3(-0.26-0.23)	16	216	0.15 (0.09~0.26)	4.36E-11	0.19 (0.10~0.36)	7.90E-07
	Quartile2(-0.52-0.26)	7	216	0.07 (0.03~0.14)	4.18E-11	0.10 (0.04~0.24)	3.55E-07
	Quartile1(<-0.52)	108	217	ref	2.91E-02	ref	
2-hydroxyacyl-CoA lyase (K12261)	Continuous scale	157	865	0.74 (0.57~0.97)	3.16E-02	0.90 (0.69~1.17)	4.90E-01
	Quartile4(>0.11)	21	216	0.25 (0.15~0.43)	3.62E-07	0.46 (0.24~0.86)	2.25E-02
	Quartile3(-0.28-0.11)	22	216	0.27 (0.16~0.44)	4.03E-07	0.43 (0.24~0.80)	1.36E-02
	Quartile2(-0.48-0.28)	31	216	0.38 (0.24~0.59)	2.27E-05	0.53 (0.30~0.94)	3.82E-02
	Quartile1(<-0.48)	83	217	ref		ref	
heterogeneous nuclear ribonucleoprotein (K15047)	Continuous scale	157	865	0.79 (0.63~1.00)	4.54E-02	0.93 (0.76~1.15)	5.22E-01
	Quartile4(>0.24)	26	216	0.25 (0.16~0.40)	6.03E-09	0.34 (0.19~0.62)	5.57E-04
	Quartile3(-0.26-0.24)	16	216	0.15 (0.09~0.27)	9.18E-11	0.20 (0.11~0.39)	2.50E-06
	Quartile2(-0.52-0.26)	10	216	0.10 (0.05~0.19)	3.91E-11	0.14 (0.06~0.29)	6.66E-07
	Quartile1(<-0.52)	105	217	ref		ref	
Fatty acid synthase subunit alpha (K00667)	Continuous scale	157	865	2.61 (1.48~4.60)	9.23E-04	3.04 (1.65~5.58)	6.01E-04
	Quartile4(>-0.07)	65	216	2.32 (1.48~3.63)	4.61E-04	1.92 (1.10~3.37)	3.97E-02
	Quartile3(-0.12-0.07)	31	215	1.11 (0.66~1.86)	6.86E-01	0.76 (0.40~1.46)	6.16E-01
	Quartile2(-0.14-0.12)	26	164	1.22 (0.71~2.11)	6.24E-01	0.86 (0.43~1.70)	6.61E-01
	Quartile1(<-0.14)	35	270	ref		ref	
Carbonic anhydrase 2 (K18245)	Continuous scale	157	865	0.61 (0.43~0.84)	2.97E-03	0.54 (0.40~0.74)	2.32E-04
	Quartile4(>0.13)	23	216	0.22 (0.14~0.36)	1.69E-09	0.16 (0.08~0.30)	2.82E-08
	Quartile3(-0.24-0.13)	15	216	0.14 (0.08~0.26)	1.06E-10	0.15 (0.07~0.30)	2.16E-07
	Quartile2(-0.46-0.24)	15	216	0.14 (0.08~0.26)	1.06E-10	0.14 (0.07~0.27)	3.23E-08
	Quartile1(<-0.46)	104	217	ref		ref	
kinesin family member 14 (K17915)	Continuous scale	157	865	0.48 (0.33~0.71)	2.24E-04	0.51 (0.37~0.72)	1.74E-04
	Quartile4(>0.13)	17	216	0.17 (0.10~0.29)	4.67E-10	0.13 (0.07~0.26)	1.24E-08
	Quartile3(-0.23-0.13)	18	216	0.18 (0.10~0.30)	3.69E-10	0.18 (0.09~0.35)	6.20E-07
	Quartile2(-0.45-0.23)	19	216	0.19 (0.11~0.31)	3.95E-10	0.17 (0.09~0.33)	2.35E-07

	Quartile1(<-0.45)	103	217	ref		ref	
GDP-perosamine N-acetyltransferase (K17939)	Continuous scale	157	865	0.43 (0.30~0.63)	1.00E-05	0.68 (0.47~1.00)	6.68E-02
	Quartile4(>0.30)	12	216	0.18 (0.09~0.34)	3.37E-07	0.34 (0.16~0.72)	8.27E-03
	Quartile3(-0.41-0.30)	34	216	0.51 (0.32~0.80)	4.81E-03	0.67 (0.38~1.18)	2.15E-01
	Quartile2(-0.56-0.41)	44	216	0.66 (0.43~1.01)	5.48E-02	0.46 (0.26~0.81)	1.07E-02
	Quartile1(<-0.56)	67	217	ref		ref	
Nonribosomal peptide synthetase protein (K16112)	Continuous scale	157	865	0.54 (0.37~0.77)	7.23E-04	0.53 (0.38~0.73)	2.20E-04
	Quartile4(>0.14)	20	216	0.20 (0.12~0.33)	6.29E-10	0.15 (0.08~0.29)	2.44E-08
	Quartile3(-0.23-0.14)	17	216	0.17 (0.10~0.29)	6.03E-10	0.15 (0.07~0.30)	1.90E-07
	Quartile2(-0.46-0.23)	18	216	0.18 (0.10~0.30)	4.78E-10	0.16 (0.08~0.32)	1.25E-07
	Quartile1(<-0.46)	102	217	ref		ref	
Tropomyosin (K17945)	Continuous scale	157	865	0.48 (0.33~0.71)	2.12E-04	0.51 (0.37~0.72)	1.78E-04
	Quartile4(>0.13)	17	216	0.17 (0.10~0.29)	3.85E-10	0.14 (0.07~0.27)	1.94E-08
	Quartile3(-0.23-0.13)	19	216	0.19 (0.11~0.32)	3.85E-10	0.19 (0.10~0.37)	1.21E-06
	Quartile2(-0.45-0.23)	19	216	0.19 (0.11~0.32)	3.85E-10	0.19 (0.10~0.36)	6.76E-07
	Quartile1(<-0.45)	102	217	ref		ref	
Enoyl-[acyl-carrier protein] reductase (K00209)	Continuous scale	157	865	1.59 (1.37~1.85)	1.79E-09	1.57 (1.32~1.86)	5.27E-07
	Quartile4(>0.11)	66	216	2.46 (1.51~3.99)	5.78E-04	2.19 (1.20~4.01)	1.96E-02
	Quartile3(-0.34-0.11)	31	216	1.15 (0.67~2.00)	6.10E-01	1.17 (0.60~2.30)	7.26E-01
	Quartile2(-0.62-0.34)	33	216	1.23 (0.71~2.11)	6.10E-01	1.01 (0.52~1.96)	9.73E-01
	Quartile1(<-0.62)	27	217	ref		ref	
Alpha-D-ribose 1-methylphosphonate (K06164)	Continuous scale	157	865	3.84 (6.0~10.09)	1.08E-05	4.47 (1.02~9.33)	6.63E-02
	Quartile4(>-0.08)	83	216	14.5 (7.17~29.57)	2.57E-13	11.47 (5.09~25.8)	8.90E-09
	Quartile3(-0.10-0.08)	45	216	7.89 (3.78~16.47)	4.95E-08	6.76 (2.91~15.68)	1.57E-05
	Quartile2(-0.10-0.10)	20	92	8.24 (3.63~18.70)	4.61E-07	6.69 (2.62~17.11)	1.08E-04
	Quartile1(<-0.10)	9	341	ref		ref	
Son of sevenless (K03099)	Continuous scale	157	865	0.77 (0.61~0.98)	3.29E-02	0.93 (0.75~1.15)	4.87E-01
	Quartile4(>0.23)	26	216	0.24 (0.15~0.39)	3.55E-09	0.36 (0.20~0.64)	8.30E-04
	Quartile3(-0.26-0.23)	16	216	0.15 (0.09~0.26)	5.59E-11	0.20 (0.10~0.38)	1.78E-06
	Quartile2(-0.52-0.26)	8	216	0.08 (0.04~0.16)	3.35E-11	0.11 (0.05~0.26)	4.61E-07
	Quartile1(<-0.52)	107	217	ref		ref	
	Continuous scale	157	865	6.41 (2.46~16.69)	1.41E-04	4.59 (1.56~13.50)	7.96E-03

	Quartile4(>-0.08)	61	216	9.69 (4.86~19.31)	1.49E-10	7.38 (3.37~16.16)	1.02E-06
Succinylornithine aminotransferase (K00840)	Quartile3(-0.11-0.08)	74	216	11.7 (5.94~23.24)	2.85E-12	10.0 (4.62~21.67)	1.15E-08
	Quartile2(-0.11-0.11)	12	90	4.57 (1.91~10.92)	6.22E-04	4.88 (1.82~13.06)	2.41E-03
	Quartile1(<-0.11)	10	343	ref		ref	
dihydrofolate reductase K07509)	Continuous scale	157	865	1.34 (1.16~1.55)	8.29E-05	0.88 (0.71~1.08)	2.22E-01
	Quartile4(>-0.14)	55	216	2.68 (1.70~4.22)	4.59E-05	0.59 (0.37~0.95)	3.02E-02
	Quartile3(-0.34-0.14)	54	214	2.65 (1.68~4.19)	3.88E-05	0.37 (0.22~0.62)	1.56E-04
	Quartile2(-0.36-0.34)	13	67	2.04 (1.03~4.06)	4.22E-02	0.63 (0.40~0.99)	4.53E-02
	Quartile1(<-0.36)	35	368	ref		ref	
Solute carrier family 10 (K14342)	Continuous scale	157	865	0.46 (0.31~0.69)	1.43E-04	0.99 (0.84~1.17)	9.17E-01
	Quartile4(>0.13)	17	216	0.16 (0.10~0.28)	1.81E-10	0.41 (0.26~0.66)	1.81E-04
	Quartile3(-0.23-0.13)	15	216	0.14 (0.08~0.26)	1.58E-10	0.17 (0.10~0.30)	2.04E-10
	Quartile2(-0.45-0.23)	21	216	0.20 (0.12~0.34)	6.96E-10	0.17 (0.09~0.30)	1.18E-09
	Quartile1(<-0.45)	104	217	ref		ref	
Dihydrofolate reductase (K18590)	Continuous scale	157	865	0.28 (0.18~0.41)	5.29E-10	0.55 (0.37~0.80)	3.25E-03
	Quartile4(>0.40)	9	216	0.09 (0.04~0.18)	9.12E-11	0.17 (0.08~0.38)	2.57E-05
	Quartile3(-0.24-0.40)	16	216	0.16 (0.09~0.28)	2.51E-10	0.31 (0.16~0.60)	9.85E-04
	Quartile2(-0.59-0.24)	31	216	0.31 (0.20~0.48)	2.11E-07	0.42 (0.24~0.72)	2.62E-03
	Quartile1(<-0.59)	101	217	ref		ref	
long-chain-fatty-acid--CoA ligase (K15013)	Continuous scale	157	865	1.96 (1.57~2.44)	1.99E-09	1.72 (1.38~2.13)	1.41E-06
	>0	65	123	4.26 (2.94~6.17)	1.73E-14	3.61 (2.23~5.87)	3.46E-07
	,0	92	742	ref		ref	
Solute carrier family 39 member 11 (K14717)	Continuous scale	157	865	0.95 (0.79~1.14)	6.11E-01	0.97 (0.79~1.19)	7.88E-01
	Quartile4(>0.05)	29	216	0.33 (0.21~0.53)	3.23E-06	0.46 (0.26~0.83)	1.19E-02
	Quartile3(-0.36-0.05)	20	216	0.23 (0.14~0.39)	7.15E-08	0.34 (0.18~0.64)	1.61E-03
	Quartile2(-0.48-0.36)	21	216	0.24 (0.15~0.40)	7.99E-08	0.23 (0.12~0.43)	1.00E-05
	Quartile1(<-0.48)	87	217	ref		ref	1.86E-02

[a]*p* value was computed using wilcoxon rank-sum test for continuous variables. [b]Quartiles of each genus were divided based on the distribution among oral cancer and controls only. [c]*p* value was computed using chi-sqaure test for continuous scale and quartiles. OR, odds ratio; CI, confidence interval; OC, oral cancer.

Table S6**.** Distribution and fold change of 15 pathways in function data set.

**Pathway**	**FC**	**log2FC**	***p*-value**[a]	**-LOG10(*p*)**
Fatty acid metabolism (ko01212)	1.276	0.352	6.96E-08	7.158
Fatty acid biosynthesis (ko00061)	1.249	0.321	1.33E-04	3.877
Microbial metabolism (ko01120)	1.311	0.390	1.90E-08	7.721
Metabolic pathways (ko01100)	0.806	-0.311	2.92E-16	15.54
Biosynthesis of secondary (ko01110)	0.880	-0.184	3.79E-08	7.421
Butanoate metabolism (ko00650)	0.864	-0.212	3.74E-05	4.427
Fatty acid elongation (ko00062)	1.220	0.287	3.85E-05	4.415
Fatty acid degradation (ko00071)	1.223	0.290	1.02E-04	3.993
Amino sugar and nucleotide sugar (ko00520)	1.080	0.111	2.67E-02	1.574
Oxidative phosphorylation (ko00190)	0.841	-0.249	5.71E-08	7.243
Degradation of aromatic (ko01220)	1.274	0.349	5.69E-11	10.25
Valine, leucine (ko00280)	1.122	0.167	1.30E-09	8.885
Nitrogen metabolism (ko00910)	0.827	-0.275	5.46E-09	8.263
Glycerophospholipid(ko00564)	1.157	0.211	2.35E-03	2.630
Arginine and proline metabolism (ko00330)	1.213	0.278	7.75E-20	19.11

15 Pathways were shown as fold change according to oral cancer and control groups. [a] *p*-value obtained as a result of wilcoxon rank-sum test of continuous variable.

Table S7**.** Logistic regression analysis of 15 pathways for oral cancer risks.

**Function**	**Logistic Regression**	**OC**	**Control**	**Univariate OR (95%CI)**	**Univariate *p*[b]**	**Multivariate OR[a] (95%CI)**	**Multivariate *p*[b]**
Fatty acid metabolism (ko01212)	Continuous scale	157	865	1.32 (1.12~1.57)	1.26E-03	1.28 (1.05~1.56)	2.24E-02
	Quartile4(>0.47)	61	216	1.43 (0.92~2.20)	1.09E-01	1.46 (0.83~2.57)	2.37E-01
	Quartile3(-0.02-0.47)	28	216	0.65 (0.39~1.09)	1.09E-01	0.63 (0.33~1.20)	2.89E-01
	Quartile2(-0.53-0.02)	25	216	0.58 (0.34~0.99)	9.16E-02	0.63 (0.33~1.23)	2.62E-01
	Quartile1(<-0.53)	43	217	ref		ref	
Fatty acid biosynthesis (ko00061)	Continuous scale	157	865	2.63 (1.49~4.64)	8.48E-04	2.73 (1.42~5.28)	4.83E-03
	Quartile4(>0.55)	54	216	1.47 (0.94~2.30)	1.87E-01	1.25 (0.70~2.21)	4.85E-01
	Quartile3(-0.00-0.55)	28	216	0.85 (0.52~1.40)	5.25E-01	0.78 (0.41~1.46)	4.85E-01
	Quartile2(-0.56-0.00)	24	216	0.72 (0.43~1.21)	2.92E-01	0.73 (0.38~1.39)	4.85E-01
	Quartile1(<-0.56)	51	217	ref		ref	
Microbial metabolism (ko01120)	Continuous scale	157	865	1.09 (0.93~1.29)	2.85E-01	1.22 (1.01~1.47)	5.44E-02
	Quartile4(>0.61)	45	216	0.94 (0.60~1.47)	7.93E-01	1.28 (0.71~2.32)	5.25E-01
	Quartile3(0.10-0.61)	37	216	0.77 (0.48~1.24)	3.80E-01	1.63 (0.89~3.01)	2.06E-01
	Quartile2(-0.52-0.10)	27	216	0.57 (0.34~0.94)	5.52E-02	0.71 (0.38~1.35)	4.45E-01
	Quartile1(<-0.52)	48	217	ref		ref	
Metabolic pathways (ko01100)	Continuous scale	157	865	0.42 (0.34~0.51)	3.24E-17	0.42 (0.33~0.54)	8.94E-12
	Quartile4(>0.79)	15	216	0.17 (0.10~0.31)	6.04E-09	0.13 (0.07~0.27)	7.73E-08
	Quartile3(0.07-0.79)	18	216	0.21 (0.12~0.36)	1.72E-08	0.28 (0.14~0.53)	2.04E-04
	Quartile2(-0.58-0.07)	37	216	0.43 (0.28~0.66)	9.98E-05	0.41 (0.23~0.72)	2.70E-03
	Quartile1(<-0.58)	87	217	ref		ref	
Biosynthesis of secondary (ko01110)	Continuous scale	157	865	0.57 (0.48~0.68)	2.76E-10	0.52 (0.42~0.66)	3.36E-08
	Quartile4(>0.72)	17	216	0.23 (0.13~0.40)	4.29E-07	0.15 (0.07~0.30)	3.89E-07

	Quartile3(0.10-0.72)	38	216	0.51 (0.33~0.79)	2.26E-03	0.48 (0.28~0.84)	1.31E-02
	Quartile2(-0.48-0.10)	27	216	0.36 (0.22~0.58)	4.14E-05	0.28 (0.15~0.52)	1.18E-04
	Quartile1(<-0.48)	75	217	ref		ref	
Butanoate metabolism (ko00650)	Continuous scale	157	865	0.91 (0.77~1.08)	3.03E-01	0.81 (0.66~1.00)	6.63E-02
	Quartile4(>0.66)	35	216	0.59 (0.37~0.93)	2.20E-02	0.38 (0.21~0.70)	3.65E-03
	Quartile3(-0.03-0.66)	28	216	0.47 (0.29~0.76)	4.55E-03	0.40 (0.21~0.75)	7.75E-03
	Quartile2(-0.61-0.03)	34	216	0.57 (0.36~0.90)	2.20E-02	0.60 (0.33~1.09)	1.22E-01
	Quartile1(<-0.61)	60	217	ref		ref	
Fatty acid elongation (ko00062)	Continuous scale	157	865	1.28 (1.08~1.51)	3.67E-03	1.51 (1.22~1.87)	2.92E-04
	Quartile4(>0.54)	53	216	1.90 (1.16~3.12)	2.19E-02	2.77 (1.47~5.24)	2.99E-03
	Quartile3(-0.13-0.54)	47	216	1.69 (1.02~2.79)	5.64E-02	2.26 (1.18~4.33)	2.04E-02
	Quartile2(-0.72-0.13)	29	216	1.04 (0.60~1.81)	8.88E-01	1.33 (0.68~2.61)	5.24E-01
	Quartile1(<-0.72)	28	217	ref		ref	
Fatty acid degradation (ko00071)	Continuous scale	157	865	1.47 (1.23~1.76)	2.02E-05	1.38 (1.10~1.73)	6.44E-03
	Quartile4(>0.42)	60	216	1.44 (0.93~2.22)	1.40E-01	1.50 (0.85~2.65)	2.85E-01
	Quartile3(-0.08-0.42)	29	216	0.69 (0.42~1.15)	1.59E-01	0.95 (0.50~1.80)	8.78E-01
	Quartile2(-0.58-0.08)	26	216	0.62 (0.37~1.05)	1.51E-01	0.86 (0.45~1.64)	7.36E-01
	Quartile1(<-0.58)	42	217	ref		ref	
Amino sugar and nucleotide sugar (ko00520)	Continuous scale	157	865	1.31 (1.11~1.54)	1.24E-03	1.07 (0.87~1.31)	5.14E-01
	Quartile4(>0.54)	62	216	1.73 (1.10~2.72)	3.48E-02	1.07 (0.59~1.91)	8.31E-01
	Quartile3(-0.09-0.54)	31	216	0.87 (0.52~1.45)	5.82E-01	0.71 (0.37~1.36)	5.47E-01
	Quartile2(-0.73-0.09)	28	216	0.78 (0.46~1.33)	4.80E-01	0.84 (0.44~1.62)	6.83E-01
	Quartile1(<-0.73)	36	217	ref		ref	
Oxidative phosphorylation (ko00190)	Continuous scale	157	865	0.68 (0.57~0.80)	8.16E-06	0.80 (0.66~0.98)	4.62E-02
	Quartile4(>0.71)	31	216	0.46 (0.29~0.73)	1.32E-03	0.63 (0.35~1.14)	2.30E-01

	Quartile3(0.04-0.71)	33	216	0.49 (0.31~0.77)	2.04E-03	0.92 (0.52~1.64)	7.81E-01
	Quartile2(-0.54-0.04)	25	216	0.37 (0.23~0.61)	1.64E-04	0.63 (0.34~1.16)	2.10E-01
	Quartile1(<-0.54)	68	217	ref		ref	
Degradation of aromatic (ko01220)	Continuous scale	157	865	1.75 (1.47~2.09)	4.94E-10	1.88 (1.49~2.38)	1.82E-07
	Quartile4(>0.56)	71	216	3.40 (2.02~5.72)	8.83E-06	5.07 (2.60~9.90)	4.51E-06
	Quartile3(-0.09-0.56)	29	216	1.39 (0.77~2.51)	2.79E-01	2.15 (1.03~4.50)	5.36E-02
	Quartile2(-0.72-0.09)	36	216	1.72 (0.97~3.05)	8.22E-02	2.35 (1.17~4.75)	2.55E-02
	Quartile1(<-0.72)	21	217	ref		ref	
Valine, leucine (ko00280)	Continuous scale	157	865	1.81 (1.51~2.17)	1.10E-10	1.62 (1.30~2.02)	2.68E-05
	Quartile4(>0.41)	73	216	2.72 (1.68~4.39)	9.00E-05	2.35 (1.28~4.31)	1.02E-02
	Quartile3(-0.11-0.41)	32	216	1.19 (0.69~2.05)	7.08E-01	1.46 (0.75~2.86)	4.03E-01
	Quartile2(-0.63-0.11)	25	216	0.93 (0.52~1.65)	8.05E-01	1.27 (0.64~2.52)	6.15E-01
	Quartile1(<-0.63)	27	217	ref		ref	
Nitrogen metabolism (ko00910)	Continuous scale	157	865	0.51 (0.42~0.62)	1.43E-11	0.52 (0.41~0.66)	7.34E-08
	Quartile4(>0.66)	17	216	0.22 (0.13~0.39)	2.45E-07	0.19 (0.09~0.37)	4.00E-06
	Quartile3(0.00-0.66)	26	216	0.34 (0.21~0.55)	1.52E-05	0.29 (0.16~0.53)	1.21E-04
	Quartile2(-0.51-0.00)	37	216	0.48 (0.31~0.75)	1.03E-03	0.46 (0.26~0.81)	9.74E-03
	Quartile1(<-0.51)	77	217	ref		ref	
Glycerophospholipid (ko00564)	Continuous scale	157	865	1.08 (0.91~1.29)	3.57E-01	1.40 (1.12~1.75)	4.37E-03
	Quartile4(>0.77)	38	216	1.12 (0.68~1.85)	6.49E-01	2.21 (1.16~4.20)	2.79E-02
	Quartile3(0.04-0.77)	46	216	1.36 (0.84~2.20)	4.23E-01	2.05 (1.10~3.82)	3.44E-02
	Quartile2(-0.73-0.04)	39	216	1.15 (0.70~1.89)	6.49E-01	1.38 (0.73~2.60)	4.20E-01
	Quartile1(<-0.73)	34	217	ref		ref	
Arginine and proline metabolism (ko00330)	Continuous scale	157	865	2.49 (2.04~3.04)	3.63E-19	1.99 (1.58~2.53)	1.72E-08
	Quartile4(>0.22)	98	216	6.15 (3.51~10.7)	4.38E-10	3.74 (1.92~7.28)	2.39E-04
	Quartile3(-0.23-0.22)	25	216	1.57 (0.82~3.02)	2.36E-01	1.38 (0.64~2.95)	5.30E-01

	Quartile2(-0.63-0.23)	18	216	1.13 (0.56~2.27)	7.32E-01	0.88 (0.38~2.02)	7.62E-01
	Quartile1(<-0.63)	16	217	ref		ref	

Logistic regression analysis was performed with standard scaled data. [a]Multivariate logistic regression was adjusted for sex, age, smoking, drinking and BMI status. [b]*p* value represents significance of the regression coefficient. OR, Odds ratio; LR, Logistic regression; OC, oral cancer; CI, confidence interval.

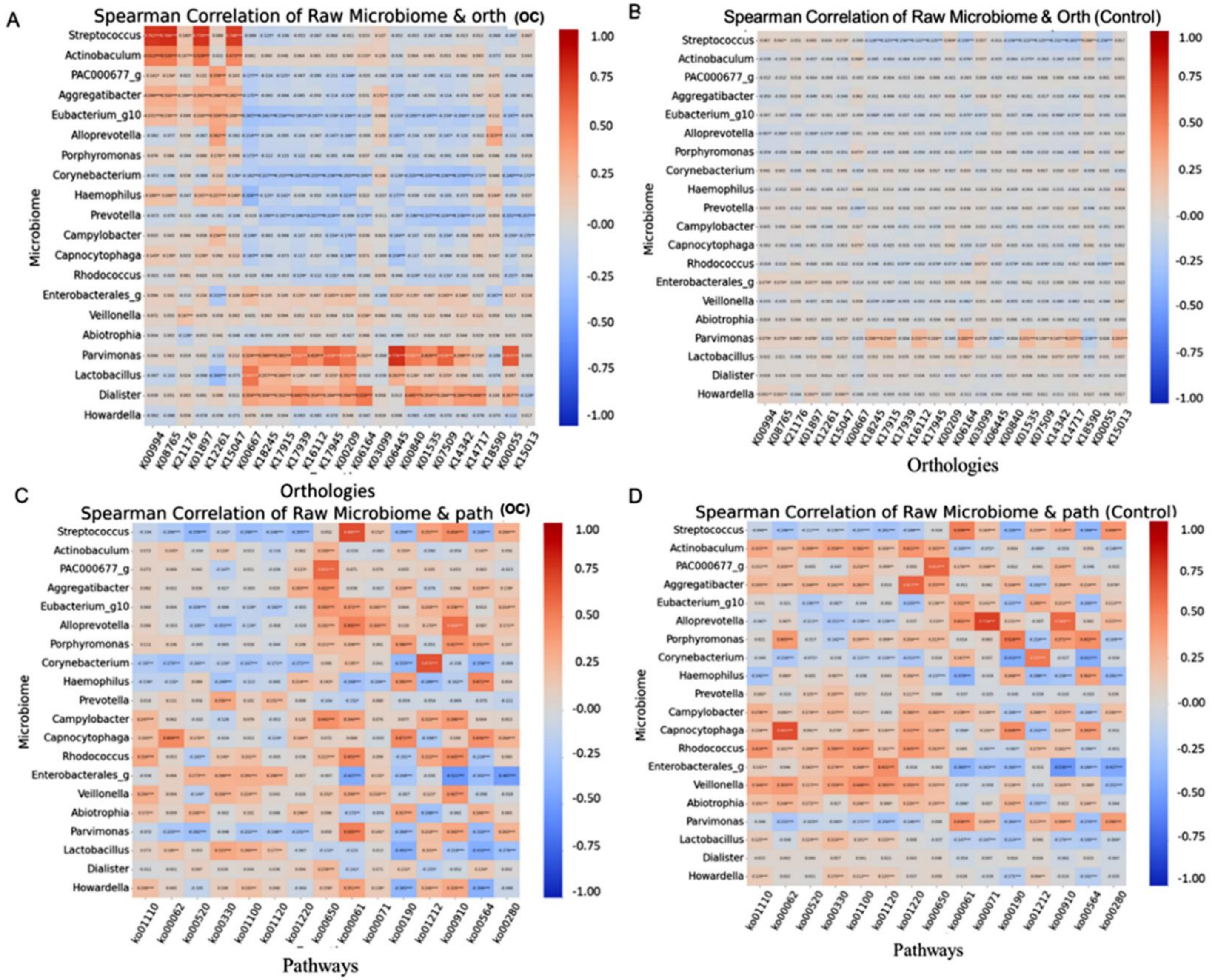

A
Spearman Correlation of Raw Microbiome & orth (OC)
B
Spearman Correlation of Raw Microbiome & Orth (Control)
Microbiome
Streptococcus
Actinobaculum
PAC000677_g
Aggregatibacter
Eubacterium_g10
Alloprevotella
Porphyromonas
Corynebacterium
Haemophilus
Prevotella
Campylobacter
Capnocytophaga
Rhodococcus
Enterobacterales_g
Veillonella
Abiotrophia
Parvimonas
Lactobacillus
Dialister
Howardella
K00994
K08765
K21176
K01897
K12261
K15047
K00667
K18245
K17915
K17939
K16112
K17945
K00209
K06164
K03099
K06445
K00840
K01535
K07509
K14342
K14717
K18590
K00055
K15013
Orthologies
1.00
0.75
0.50
0.25
-0.00
-0.25
-0.50
-0.75
-1.00
C
Spearman Correlation of Raw Microbiome & path (OC)
D
Spearman Correlation of Raw Microbiome & path (Control)
ko01110
ko00062
ko00520
ko00330
ko01100
ko01120
ko01220
ko00650
ko00061
ko00071
ko00190
ko01212
ko00910
ko00564
ko00280
Pathways

Figure S4. Spearman correlation heatmap between microbiome and function data (A, B) correlation between 20 genera and 24 orthologs (Carnitine O-palmitoyltransferase 1(K08765), Acyl-CoA dehydrogenase (K06445), Long-chain acyl-CoA synthetase (K01897), Diacylglycerol choline phosphotransferase (K00994), H+-transporting ATPase (K01535)) in oral cancer and control group, (C,D) correlation between 20 genera and 15 pathways (Fatty acid biosynthesis (ko00061), Fatty acid metabolism (ko01212)) in cancer and control, (The color chart range has been set from -1.00 to 1.00.)

Supplementary Protocol S1: CD4+ Immunohistochemistry (IHC)

Protocol Materials:

- **Tissue Sections:** 4 µm-thick paraffin-embedded tissue sections.
- **Deparaffinization Solution:** Xylene.
- **Rehydration Solutions:** Ethanol series (100%, 95%, 70%) and distilled water.
- **Antigen Retrieval Solutions:**
  - Tris-EDTA buffer (pH 9.0).
  - Sodium citrate buffer (pH 6.0).
- **Blocking Solution:** 3% hydrogen peroxide (H_2O_2) in distilled water.
- **Primary Antibody:** Anti-CD4 antibody (dilution according to manufacturer's instructions).
- **Secondary Antibody:** HRP-conjugated secondary antibody (dilution according to manufacturer's instructions).
- **DAB Substrate:** 3,3'-diaminobenzidine (DAB) substrate for visualizing the antibody.
- **Counterstain:** Hematoxylin.
- **Mounting Media:** Neutral mounting medium for microscope slides.

Procedure:

1. **Sectioning:**
   - Cut paraffin-embedded tissue samples into 4 µm-thick sections using a microtome.
   - Mount the sections onto glass slides.
2. **Deparaffinization:**
   - Immerse the slides in xylene (2–3 changes, 5 minutes each) to remove paraffin.
3. **Rehydration:**
   - Sequentially rehydrate the slides by passing through decreasing concentrations of ethanol:
     - 100% ethanol for 5 minutes.
     - 95% ethanol for 5 minutes.
     - 70% ethanol for 5 minutes.
   - Rinse the slides in distilled water.
4. **Antigen Retrieval:**
   - Heat the slides in Tris-EDTA buffer (pH 9.0) or sodium citrate buffer (pH 6.0) using a microwave or pressure cooker for 20–30 minutes.
   - Allow the slides to cool to room temperature in the buffer.
   - Rinse the slides in distilled water.

5. **Blocking Endogenous Peroxidases:**
    - Incubate the tissue sections in 3% hydrogen peroxide (H_2O_2) for 10 minutes to block endogenous peroxidase activity.
    - Rinse the slides in distilled water.
6. **Primary Antibody Incubation:**
    - Block non-specific binding sites using a blocking serum or protein block (optional).
    - Incubate the tissue sections with the primary antibody against CD4 (diluted according to manufacturer's instructions) for 1–2 hours at room temperature or overnight at 4°C.
    - Rinse the slides in phosphate-buffered saline (PBS) or Tris-buffered saline (TBS).
7. **Secondary Antibody Incubation:**
    - Incubate the tissue sections with an HRP-conjugated secondary antibody (diluted according to manufacturer's instructions) for 30–60 minutes at room temperature.
    - Rinse the slides in PBS or TBS.
8. **DAB Development:**
    - Apply the DAB substrate to the slides and monitor for color development under a microscope (2–10 minutes).
    - Stop the reaction by rinsing the slides in distilled water.
9. **Counterstaining:**
    - Counterstain the tissue sections with hematoxylin for 30 seconds to 1 minute.
    - Rinse in tap water to remove excess hematoxylin.
    - Dip slides in 70% ethanol, followed by 95% ethanol to remove excess water.
10. **Dehydration and Mounting:**
    - Dehydrate the sections by passing through 100% ethanol (2 changes, 5 minutes each).
    - Clear the slides in xylene (2–3 changes, 5 minutes each).
    - Mount the tissue sections with a neutral mounting medium.

Notes:

- Ensure that all reagents are prepared fresh, and follow safety guidelines while handling chemicals.
- Adjust antibody dilutions based on specific experimental needs and manufacturer recommendations.
